# Bisphenol S impairs oocyte quality by inducing gut microbiota dysbiosis

**DOI:** 10.1128/msystems.00912-24

**Published:** 2024-12-20

**Authors:** Jiaming Zhang, Xiaoxia Yu, Weidong Li, Yunjing Jiang, Liangran Zhang, Shunxin Wang

**Affiliations:** 1 State Key Laboratory of Reproductive Medicine and Offspring Health, Center for Reproductive Medicine, Institute of Women, Children, and Reproductive Health, Shandong University, Jinan, China; 2 Advanced Medical Research Institute, Shandong University, Jinan, Shandong, China; 3 Center for Cell Structure and Function, Shandong Provincial Key Laboratory of Animal Resistance Biology, College of Life Sciences, Shandong Normal University, Jinan, Shandong, China; 4 National Research Center for Assisted Reproductive Technology and Reproductive Genetics, Shandong University, Jinan, Shandong, China; 5 Key Laboratory of Reproductive Endocrinology (Shandong University), Ministry of Education, Jinan, Shandong, China; 6 Shandong Technology Innovation Center for Reproductive Health, Jinan, Shandong, China; 7 Shandong Provincial Clinical Research Center for Reproductive Health, Jinan, Shandong, China; 8 Shandong Key Laboratory of Reproductive Research and Birth Defect Prevention, Jinan, Shandong, China; Chinese Academy of Sciences, Beijing, China

**Keywords:** bisphenol S, oocyte, gut microbiota, alginate oligosaccharide

## Abstract

A good quality egg is essential for a successful pregnancy and early embryo development. Oocyte development is vulnerable to environmental exposures. Bisphenol S (BPS) is widely used as a replacement for its analog bisphenol A, but the reproductive toxicity of BPS has been of great concern. In this study, we showed that BPS exposure induces dysbiosis of the gut microbiota, which further leads to intestinal permeability and inflammation, and ultimately impairs oocyte quality. More importantly, we found that alginate oligosaccharide reshapes the gut microbiota to improve gut homeostasis, thereby preventing the deleterious effects of BPS on the gut and oocytes. Overall, this study not only demonstrates that BPS exposure impairs the intestine and oocytes by inducing dysbiosis of the gut microbiota but also develops a preventive strategy.

**IMPORTANCE:**

Oocyte development is vulnerable to stimulation by intrinsic and extrinsic factors, particularly many environmental pollutants and chemicals in daily life. The reproductive toxicity of bisphenol S has been of great concern, although it is widely used as a safe substitute for its analog bisphenol A. However, it is not known how bisphenol S impairs oocyte quality. This work presents the exciting finding that bisphenol S induces gut microbiota dysbiosis, which further leads to increased intestinal permeability and inflammation and ultimately damages oocytes. More importantly, we show that alginate oligosaccharide improves gut homeostasis by reshaping the gut microbiota, therefore preventing the bisphenol S-induced gut microbiota dysbiosis and gut and oocyte damage. These findings present a major advance in the understanding of bisphenol S toxicity to oocytes and also provide a preventive strategy.

## INTRODUCTION

Infertility is a global problem, affecting ~15% of couples of reproductive age. About half of infertility is attributed to women. Oocyte quality is a critical determinant of pregnancy and early embryonic development (1). Poor oocyte quality has been recognized as a common cause of infertility and spontaneous abortion in humans (2).

Male meiosis is a successive process to produce sperm. Female meiosis, however, begins in the embryo and is arrested at the diplotene stage of meiotic prophase I before birth for many years. During each menstrual or estrus cycle, only one or a few fully grown oocytes resume meiosis and develop into mature oocytes capable of fertilization and embryo development (1). During this super-long meiotic process, oocytes are susceptible to stimulation by intrinsic and extrinsic factors, especially many environmental pollutants and chemicals in daily life.

Bisphenol S (BPS) is chemically more stable than its analog bisphenol A (BPA) and has therefore been widely used in many common consumer products to replace BPA to reduce its harmful effects on human health. BPS is frequently detected in the environment, including air, water, and food (3). Daily exposure doses of BPS can reach ~1.7 µg/day for the general population, and ~22 µg/day for occupationally exposed individuals (4). BPS enters the human body via the same routes of exposure as BPA, for example, ingestion, inhalation, and dermal contact (5). The concentration of BPS is detected up to 10 ng/mL in body fluids, urine, serum, and follicular fluid (6, 7).

Epidemiological studies have shown that BPS exposure is strongly associated with many diseases, such as hypertension and reduced semen quality (8, 9). The reproductive toxicity of BPS has been of great concern. Studies have shown that BPS has various adverse impacts on both male and female fertility. BPS exposure can damage the testicular and ovarian architecture, impair sperm and oocyte maturation and development, and affect their quality and quantity (10, 11).

Given the similarities between BPS and BPA in their structures and adverse effects, it has been proposed that BPS acts as a xenoestrogen and interacts with receptor-mediated signaling pathways to disrupt endocrine (12). However, some studies have also shown that BPS does not affect estrogen receptors and therefore acts differently to BPA (13). Many studies show that BPS-induced defects are usually accompanied by oxidative stress, mitochondrial damage, and apoptosis, and thus, it seems that these defects may be caused by the mitochondrial oxidative stress due to imbalanced ROS generation and elimination (14, 15). However, it is unclear whether this hypothesis is correct and how BPS induces oxidative stress. It is also possible that different concentrations of BPS act through different pathways in different cell/organism types.

It is widely accepted that the gut microbiome is important for human health. Several studies have shown that exposure to BPA and its analogs causes gut microbiota dysbiosis and affects gut function and metabolites, for example, increases intestinal permeability and triggers an inflammatory response (16). There are also studies showing that changes in gut microbes affect reproductive health and fertility, for example, fecal microbiota transplantation (FMT) from women with polycystic ovary syndrome leads to disruption of ovarian function and infertility in recipient mice (17). However, it is unclear whether BPS impairs oocyte quality by interfering with the gut microbiota.

Here, we have shown that BPS exposure leads to gut microbiota dysbiosis and intestinal inflammation, ultimately impairing oocyte quality. We also showed that alginate oligosaccharide (AOS) can prevent the deleterious effects of BPS on gut microbes to protect oocytes. Thus, this study also provides a preventive strategy for BPS-induced damage.

## RESULTS

### BPS exposure impairs oocyte quality

**Fig 1:**
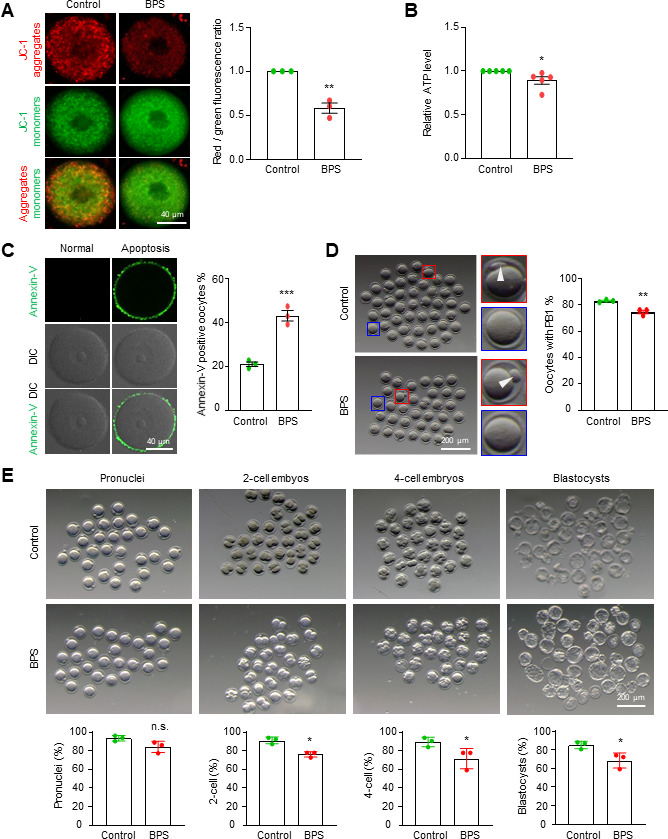
BPS treatment reduces oocyte quality *in vivo*. (**A**) Representative images of JC-1 assay and quantification of fluorescence intensity to show the relative MMP in GV oocytes. MMP was calculated as the ratio of red-to-green fluorescence intensity. (**B**) The relative ATP content in GV oocytes as determined by the firefly luciferase assay. (**C**) Representative images showing annexin V staining of GV oocytes and quantification to show the percentage of early apoptotic oocytes. (**D**) Representative images showing PB1 extrusion (white arrowhead) and quantification to show the percentage of oocytes with PB1. (**E**) MII oocytes were obtained from treated mice, and IVF was performed. Representative images and the quantification to show oocytes with pronuclei, two-cell and four-cell embryos, and blastocysts. The rates of pronuclei, two-cell embryos, four-cell embryos, and blastocysts were calculated based on the initial number of MII oocytes. Error bars, SEM from three (**A, C–E**) or five (**B**) independent experiments. Approximately 25 GV oocytes from two mice per treatment in each experiment. Totally, 78 and 76 GV oocytes were used for control and BPS treatment, respectively (**A**). Ten GV oocytes from one mouse per treatment in each experiment. Totally, 50 GV oocytes from five mice were used for control and BPS treatments, respectively (**B**). In addition, ~30 GV oocytes from two mice per treatment in each experiment. Totally, 85 and 88 GV oocytes for control and BPS treatments, respectively (**C**); ~35 GV oocytes from two mice per treatment in each experiment. Totally, 115 and 95 GV oocytes for control and BPS treatments, respectively (**D**); and ~30 MII oocytes from one mouse per treatment in each experiment. Totally, 94 and 86 MII oocytes for control and BPS treatments, respectively (**E**). n.s., *P* ≥ 0.05; *, *P* < 0.05; **, *P* < 0.01; Student’s *t*-test.

To investigate whether BPS impairs oocytes by inducing gut microbial dysbiosis, mice were fed with corn oil (control) or 1 mg/kg bw BPS (dissolved in corn oil) by daily intragastric gavage for 4 weeks (Materials and Methods). Fully grown GV oocytes were collected from mouse ovaries, and mitochondrial membrane potential (MMP) was assessed using the JC-1 (a lipophilic cationic fluorescent dye) assay. JC-1 monomers, which exhibit green fluorescence, can enter mitochondria with high MMP and form aggregates, which exhibit red fluorescence. Therefore, the ratio of red-to-green fluorescence intensity indicates the relative MMP level in the cells. MMP levels in oocytes from BPS-treated mice were moderately decreased to 66.4% of that in the control (Fig. 1A). This suggests that BPS exposure impairs oocyte mitochondria. Consistent with this, ATP levels, as measured by the firefly luciferase assay, were decreased to 89.4% of control (Fig. 1B). Damaged mitochondria often lead to apoptosis, and as expected, an increased frequency of early apoptotic oocytes was observed by annexin-V staining, which recognizes and binds phosphatidylserine exposed on the outside surface from the inner membrane surface with high affinity (Fig. 1C). Furthermore, the percentage of oocytes with an extruded first polar body (PB1), an important criterion for oocyte development, was slightly but significantly decreased from 83.0% in the control to 74.7% in BPS-treated mice after *in vitro* maturation for 12 h (Fig. 1D). These results indicate reduced oocyte quality in BPS-treated mice.

To further investigate the developmental potential of the oocytes, cumulus-oocyte complexes were collected from the oviductal ampulla of superovulated mice, and *in vitro* fertilization (IVF) assays were performed. The results showed that the metaphase II (MII) oocytes from BPS-treated mice had reduced fertilization capacity (from 93.5% to 84.1%) and reduced embryo development potential to give rise to blastocysts (from 85.1% to 68.5%) (Fig. 1E). These results are consistent with previous reports and confirm that BPS exposure impairs oocyte quality, although it does not affect ovarian or body weight (Fig. S1A and B) (10).

### BPS impairs oocyte quality by inducing gut microbiota dysbiosis

**Fig 2:**
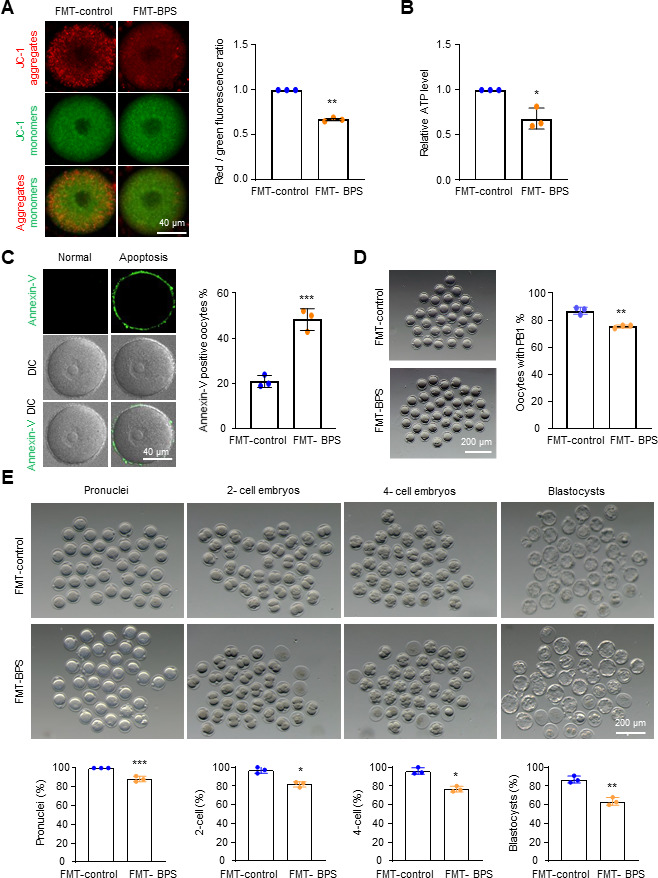
FMT from BPS-treated mice impairs oocyte quality. (**A**) Representative images and the quantification of MMP in GV oocytes by JC-1 assay. (**B**) Quantification of relative ATP content in GV oocytes by the firefly luciferase assay. (**C**) Representative images showing early apoptotic oocytes by annexin V staining and quantification to show the percentage of early apoptotic oocytes. (**D**) Representative images and the quantification to show the percentage of oocytes with PB1 extrusion. The rates of pronuclei, two-cell embryos, four-cell embryos, and blastocysts were calculated based on the initial number of MII oocytes. (**E**) Representative images and the quantification to show oocytes with pronuclei, two-cell and four-cell embryos, and blastocysts. Error bars, SEM from three independent experiments (**A–E**). Approximately 20 GV oocytes from two mice per treatment in each experiment. Totally, 65 and 64 GV oocytes were used for FMT-control and FMT-BPS treated mice, respectively (**A**). Ten GV oocytes from one mouse was used per treatment in each experiment. Totally, 30 GV oocytes for FMT-control and FMT-BPS treated mice, respectively (**B**); ~30 GV oocytes from two mice were used per treatment in each experiment. Totally, 88 and 83 GV oocytes were used for FMT-control and FMT-BPS treated mice, respectively (**C**), and ~30 GV oocytes from two mice per treatment in each experiment. Totally, 96 and 95 GV oocytes for FMT-control and FMT-BPS treated mice, respectively (**D**); ~30 MII oocytes from one mouse per treatment in each experiment. Totally, 90 and 92 MII oocytes were used for FMT-control and FMT-BPS treated mice, respectively (**E**). *, *P* < 0.05; **, *P* < 0.01; ***, *P* < 0.001; Student’s *t*-test.

To explore whether BPS exposure impairs oocytes by disrupting the gut microbiota, microbiota were collected in fecal samples from BPS-treated or control mice and transferred to the ICR female recipient mice by daily gavage for 4 weeks. Fully grown GV oocytes were then collected from the above recipient mice (FMT-BPS for FMT from BPS treated mice and FMT-control for FMT from corn oil treated mice). For GV oocytes from FMT-BPS mice, the MMP and ATP levels were decreased, the frequency of apoptosis was increased, the frequency of PB1 extrusion was decreased, and the fertilization capacity of the oocytes and the potential for the blastocyst development were also decreased compared with those in FMT-control (Fig. 2). Moreover, the alterated levels of these parameters examined in FMT-BPS treated mice were comparable with those of BPS directly treated mice (compare each panel in Fig. 2 with the corresponding panel in Fig. 1). Importantly, these defects in oocytes were not caused by the residual BPS in fecal samples (Fig. S2). These results support the idea that BPS impairs oocytes by inducing dysbiosis of the gut microbiota.

The gut microbiota consists mainly of bacteria (18). To investigate the possible changes in bacterial diversity in BPS-treated and FMT-BPS-treated mice compared with the corresponding controls, 16S rDNA sequencing was performed with enough coverage depth. (Fig. S3A). Consistent with previous reports, the mouse intestinal microbiota is mainly composed of Firmicutes, Bacteroidetes, Proteobacteria, and Actinobacteria at the phylum level, and *Muribaculaceae*, *Bacteroides*, *Lachnospiraceae_NK4A136_group*, *Lachnospiracceae_FCS020_group*, *Roseburia*, *Alistipes*, *Blautia*, *Lachnoclostridium*, *Colidextribacter*, *Parasutterella*, *Mucispirillum*, *Oscillibacter*, *Lactobacillus*, and *Dubosiella* at the genus level (Fig. S3B and C) (19).

**Fig 3:**
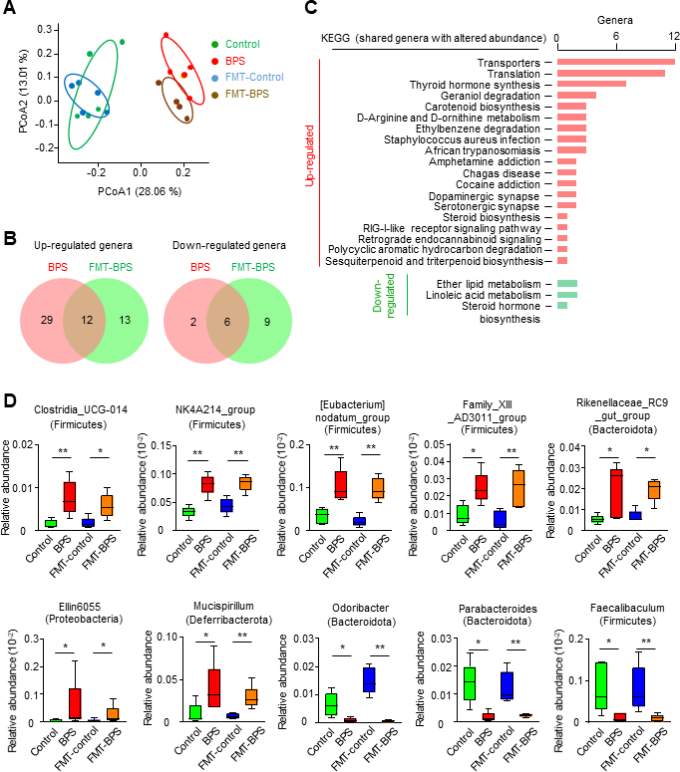
BPS and FMT-BPS treatments have similar effects on the gut microbiota. (**A**) Principal coordinate analysis (PCoA) of 16S rDNA data based on the Bray-Curtis distance. (**B**) Venn plot showing upregulated (increased abundance) and downregulated (decreased abundance) genera (*P* < 0.05 and LDA ≥ 2) in BPS and FMT-BPS treated mice. (**C**) KEGG analysis of upregulated and downregulated genera shared between BPS and FMT-BPS in (**B**), respectively. (**D**) Representative examples of upregulated and downregulated genera shared between BPS and FMT-BPS treated mice. Error bars, SEM from five independent experiments; one mouse per treatment in each experiment. *, *P* < 0.05; **, *P* < 0.01; Wilcoxon rank-sum test.

Although the Shannon index (α diversity) of the microbiota did not show any significant difference between the different experimental groups, a Bray-Curtis distance-based principal coordinate analysis (PCoA) of the microbiota structure (β diversity) clearly showed that the gut bacteria from BPS-treated and FMT-BPS-treated mice were closely clustered and obviously different from the two sets of controls, which were also closely clustered (Fig. 3A; Fig. S3D and E). This suggests that both BPS and FMT-BPS treatments cause similar changes in gut bacteria. Further analysis showed that BPS-treated mice and FMT-BPS recipient mice shared 12 upregulated (increased abundance) and six downregulated (decreased abundance) bacterial genera (*P* < 0.05 and linear discriminant analysis [LDA] ≥ 2 by LEfSe analysis [LDA effect size]) (Fig. 3B; Table S1). These shared differential bacterial genera probably affect oocyte quality. The KEGG analysis identified upregulated pathways related to inflammation and hormonal regulation, including *Staphylococcus aureus* infection, African trypanosomiasis, Chagas disease, steroid biosynthesis, and RIG-I-like receptor signaling pathway (Fig. 3C; Table S2). KEGG analysis also identified the downregulated steroid hormone biosynthesis pathway, which also plays an important role in regulating inflammation and immunity (Fig. 3C; Table S2 [20]). Among the upregulated differential bacteria, at least seven genera have been reported to have deleterious effects: *Clostridia_UCG-014, NK4A214_group*, *[Eubacterium]_nodatum_group*, and *Family_XIII_AD3011_group* (belonging to the phylum Firmicutes), *Rikenellaceae_RC9_gut_group* (Bacteroidota), *Ellin6055* (Proteobacteria), and *Mucispirillum* (Deferribacterota) (Fig. 3D; Table S1) (21–25). Increased abundance of each of these genera is associated with several diseases. Interestingly, these bacteria are all closely associated with inflammatory responses (21–25), or example, more Firmicutes can increase intestinal permeability and ultimately leads to elevated levels of pro-inflammatory factors such as IL-6 and TNF-α (26). Among the downregulated differential bacteria, at least three genera function in anti-inflammation and are considered beneficial bacteria: *Odoribacter* and *Parabacteroides* belong to the phylum Bacteroidota, and *Faecalibaculum* belongs to the phylum Firmicutes (Fig. 3D; Table S1) (27). Taken together, BPS and FMT-BPS treatments lead to common detrimental changes in the gut microbiota, specifically, more pro-inflammatory bacteria and fewer anti-inflammatory bacteria, which would induce gut inflammation.

### BPS and FMT-BPS induce intestinal inflammation to impair oocytes

**Fig 4:**
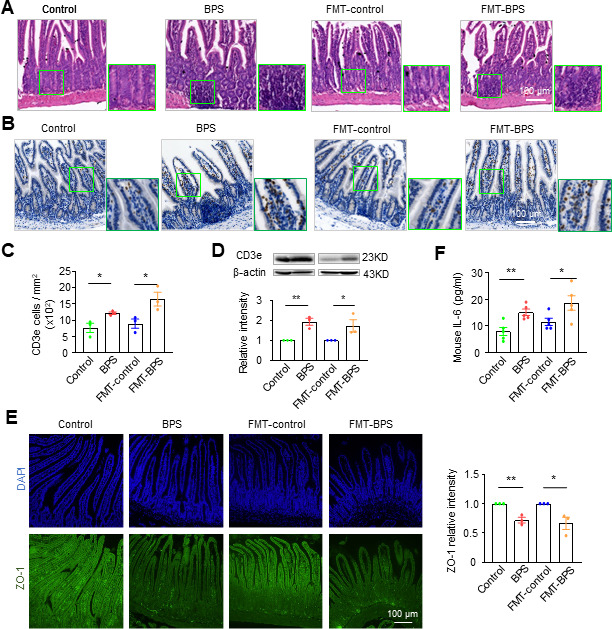
BPS and FMT-BPS induced intestinal inflammation. (**A**) HE staining of intestinal sections showing increased intestinal lymphocyte infiltration in BPS-treated and FMT-BPS mice. Scale bar, 100 µm. (**B**) Immunostaining for CD3e in intestinal sections. Scale bar, 100 µm. (**C**) Quantification of CD3e cell density in (**B**) (**D**) Western blot and the quantification to show CD3e protein abundance in the intestine. (**E**) Immunostaining of ZO-1 (green) in intestinal sections and the quantification of the relative fluorescence intensity of ZO-1. DNA was counterstained with DAPI (blue). Scale bar = 100 µm. (**F**) Quantification of interleukin-6 in blood by ELISA. Error bars, SEM from three independent experiments (**A–E**) and five independent experiments (**F**); one mouse per treatment in each experiment. *, *P* < 0.05, **, *P* < 0.01; Student’s *t*-test.

To explore whether these BPS-treated and FMT-BPS-treated mice have increased intestinal inflammation and systemic chronic inflammation, we examined the inflammatory state of the small intestine and pro-inflammatory cytokines in the blood. HE staining of intestinal sections showed increased intestinal lymphocyte infiltration in BPS-treated and FMT-BPS-treated mice (Fig. 4A). CD3e is a T-cell surface marker. CD3e-positive cells were counted in intestinal sections by immunohistochemical staining, and CD3e protein from intestinal extracts was quantified by western blot. The results showed a significant increase in CD3e level in both BPS-treated and FMT-BPS-treated mice (Fig. 4B through D). Intestinal permeability can be assessed by the levels of intestinal adhesion proteins, such as ZO-1. When examined by immunostaining, a significant reduction (~30%) in ZO-1 levels was observed in BPS-treated and FMT-BPS-treated mouse intestinal sections, indicating increased intestinal permeability (Fig. 4E). Increased intestinal permeability can lead to lipopolysaccharide infiltration as well as activation of the Toll-like receptor signaling pathway, resulting in the release of pro-inflammatory cytokines (26). As expected, blood interleukin-6 (IL-6, a pro-inflammatory factor) increased by more than 60% in BPS-treated and FMT-BPS-treated mice (Fig. 4F). Previous studies have shown that *in vitro* treatment of oocytes with IL-6 can cause spindle abnormalities and reduce oocyte quality (28). Increased IL-6 is also closely associated with abnormal ovarian lipid metabolism and reduced oocyte quality in endometriosis patients, PCOS patients, and obese women (29). Therefore, our results suggest that dysbiosis of the gut microbiota induces elevated intestinal permeability and inflammation, which further leads to elevated pro-inflammatory cytokines (e.g., IL-6) in the blood.

**Fig 5:**
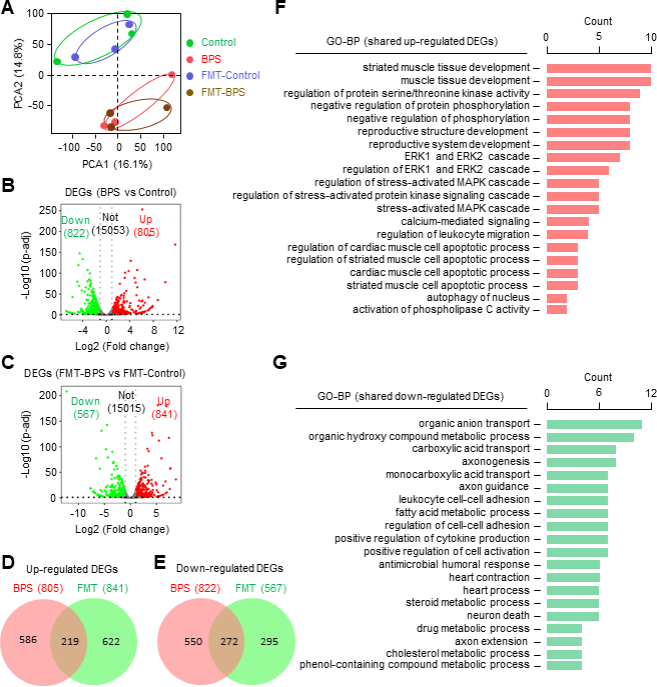
RNA-seq analyses of oocytes from BPS and FMT-BPS-treated mice. (**A**) PCA of RNA-seq data. *n* = 3 independent experiments. Twenty GV oocytes from two mice per treatment for each experiment. (**B**) Volcano plots showing DEGs in BPS-treated oocytes. (**C**) Volcano plots showing DEGs (*P* < 0.05 and |log2(fold change)| ≥ 1) in FMT-BPS-treated oocytes. Red dots, upregulated DEGs; green dots, downregulated DEGs; gray dots, not significantly changed. (**D and E**) Venn plots showing upregulated (**D**) and downregulated (**E**) DEGs in both BPS and FMT-BPS oocytes. (**F**) GO enrichment analysis of upregulated DEGs in both BPS and FMT-BPS treatments. (**G**) GO enrichment analysis of downregulated DEGs in both BPS and FMT-BPS treatments.

To further investigate how BPS impairs oocytes and whether this is related to inflammatory responses, RNA-seq was performed with oocytes from BPS-treated, FMT-BPS-treated mice, and their corresponding control mice (Fig. 5). As expected, the principal component analysis (PCA) revealed that the oocyte transcriptomes of BPS-treated and FMT-BPS-treated mice were closely clustered and separated from the two different sets of controls, which were also closely clustered (Fig. 5A). There were 805 upregulated genes and 822 downregulated genes in the oocytes from BPS-treated mice compared with those from control mice (Fig. 5B). Similarly, 841 genes were upregulated and 567 genes were downregulated in the oocytes from FMT-BPS treated mice compared with those from FMT-control mice (Fig. 5C). Further analysis showed that aproximately one-third of the upregulated DEGs (differentially expressed genes) were shared between BPS-treated and FMT-BPS-treated mouse oocytes (Fig. 5B through D). Similarly, approximately one-third of the downregulated DEGs were shared between the two types of treatment (Fig. 5B, C, and E).

GO-BP analysis of the shared upregulated DEGs in BPS-treated and FMT-BPS-treated mouse oocytes revealed that enrichment of biological processes associated with the negative regulation of the ERK cascade, the stress-activated-MAPK cascade (JNK signaling pathway), and apoptosis (Fig. 5F). The stress-activated-MAPK cascade is involved in many cellular processes, including cell proliferation, differentiation, apoptosis, and inflammatory responses (30). ERK signaling has important functions in oocyte meiosis and the development of follicles and early embryos (31). We noted that the expression of *Dusp1* was greatly upregulated (6.5-fold), which could promote the MAPK pathway towards apoptosis while suppressing the ERK signaling pathway (30). Moreover, many upregulated genes, belonging to protein serine/threonine kinase activity and stress-activated protein kinase signaling cascade, can also suppress the ERK pathway (32). Additionally, the upregulated expression of *Nox4* in the ERK pathway can increase the level of ROS (33). These results suggest that both BPS and FMT-BPS treatments cause intestinal inﬂammation, which may further activate the MAPK signal transduction pathway to suppress the ERK signaling pathway and ultimately impair oocytes.

GO-BP analysis of the shared downregulated DEGs in BPS-treated and FMT-BPS-treated mouse oocytes showed that the enriched biological processes were mainly associated with cell-cell adhesion, cell activation, material transport, positive regulation of cytokine production, and steroid metabolic processes (Fig. 5G). C-C motif chemokine ligand 2 (*Ccl2*) and tenascin R (*Tnr*), key proteins mediating cell-cell adhesion, were greatly downregulated (3-fold to 5-fold) (34, 35). In addition, the key factors mediating the transport of nutrients and metabolites, including *Stard10, Cftr, Lrp2, Abcb11*, and *Emb*, were downregulated by 2-fold to 5-fold (36–41). This suggests a possible reduced communication of signaling (e.g., steroid hormone) and nutrients between the oocyte and the surrounding granulosa and a reduced immune response in the oocyte. These alterations, in combination with the upregulated biological processes, suggest a coherent regulatory pathway: increased intestinal inflammation and impaired communication between granulosa and oocyte activate the MAPK signaling pathway and related pathways that ultimately impairing the oocyte.

Interestingly, GO-BP analysis of DEGs specifically occurring in BPS-treated mouse oocytes showed that most DEGs were involved in processes associated with reduced immune response (Fig. S4A and B). However, FMT-BPS-specific DEGs were associated with various biological processes including cytoskeletal organization, lipid transport, and also immune response (Fig. S4C and D). These results suggest that BPS and FMT-BPS treatments may also have different effects on oocytes, respectively. It is also possible that the difference is caused by different treatments: BPS would gradually alter the gut microbiota and FMT-BPS would directly deliver the altered gut microbiota, and the consequence is likely caused by different duration of altered microbiota and different dosage effects.

Taken together, our analyses on BPS- and FMT-BPS-treated mice suggest that BPS exposure induces gut microbiota dysbiosis and inflammation, which impairs communications between granulosa and oocyte and ultimately impairs oocytes.

### AOS prevents/rescues BPS-induced gut microbial dysbiosis and protects oocytes

**Fig 6:**
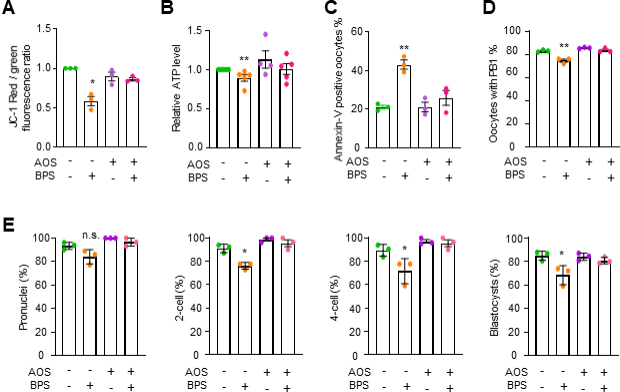
AOS prevents/recues BPS-induced oocyte damage. (**A**) The levels of MMP in GV oocytes from indicated treatments by JC-1 assay. (**B**) Relative ATP levels in GV oocytes from indicated treatments by the firefly luciferase assay. (**C**) The percentage of early apoptotic oocytes by annexin V staining. (**D**) The percentage of oocytes with PB1 extrusion. (**E**) The percentage of oocytes with pronuclei, two-cell and four-cell embryos, and blastocysts after *in vitro* fertilization. The rates of pronuclei, two-cell embryos, four-cell embryos, and blastocysts were calculated based on the initial number of MII oocytes. Error bars, SEM from three independent experiments. Approximately 25 GV oocytes from two mice per treatment in each experiment. Totally, 78, 76, 73, and 84 GV oocytes for different treatments, respectively (**A**). Ten GV oocytes from one mice per treatment in each experiment. Totally, 50 GV oocytes for different treatments, respectively (**B**). Approximately 25 GV oocytes from two mice per treatment in each experiment. Totally, 85, 88, 87, and 77 GV oocytes for different treatments, respectively (**C**); ~30 GV oocytes from two mice per treatment in each experiment. Totally, 115, 95, 85, and 86 GV oocytes for different treatments, respectively (**D**); and ~30 MII oocytes from one mouse per treatment in each experiment. Totally, 94, 86, 78, and 84 MII oocytes for control and BPS treatments, respectively (**E**). n.s., *P* ≥ 0.05; *, *P* < 0.05; **, *P* < 0.01; Student’s *t*-test. Data for control (no BPS/AOS) and BPS only were taken from the corresponding data in Fig. 1.

Alginate oligosaccharide (AOS) is derived from alginate with superior biological activities and therapeutic potential. For example, AOS shows anti-inflammatory, antimicrobial, and antioxidant activities and can regulate immune responses and reduce hypertension and blood glucose levels (42). We wondered whether AOS could prevent/rescue BPS-induced oocyte damage. For this purpose, female mice were fed with AOS (10 mg/kg bw) alone or AOS in combination with BPS (1 mg/kg bw BPS and 10 mg/kg bw AOS) (simply as AOS/BPS) by daily gavage for 4 weeks. Feeding AOS alone or AOS/BPS did not have any obvious effect on the body weight or ovarian weight of the mice (Fig. S1). When oocytes from AOS-only treated mice were examined, there was no difference in oocyte quality compared with the untreated control (Fig. 6). However, when compared with oocytes from BPS-treated mice, the quality of oocytes from AOS/BPS-treated mice was obviously improved and comparable with AOS alone or untreated control (Fig. 6). This was seen at the levels of MMP, ATP, early apoptosis, PB1 extrusion, and the potential of fertilization and blastocyst development (Fig. 6). Therefore, AOS feeding can prevent/rescue BPS-induced damage to oocytes.

To investigate how AOS prevents or rescues BPS-induced damage, 16S rDNA sequencing of the gut microbiota was performed, and PCoA analysis showed that the gut mcirobiota from AOS-treated and BPS/AOS-treated mice were well separated from the untreated and BPS-treated mice, although the Shannon indices showed no difference (Fig. S3D and E). The structural composition of the gut microbiota was significantly altered in AOS-treated mice as previously reported (43): some beneficial genera (*Romboutsia*, *Bifidobacterium*, *Lachnospiraceae_UCG_004*, *Butyricimonas*) and harmful genera (*Bilophila*, *Clostridia_UCG_014*, *Legionella*, *Tyzzerella*) were more abundant, whereas some other beneficial genera (*Parabacteroides*, *Odoribacter*, *Prevotellaceae_UCG_001*) and harmful genera (*Mycoplasma*) were less abundant (Fig. S5A). KEGG analysis performed with PICRUSt2 confirmed these changes: some beneficial pathways (e.g., unsaturated fatty acid biosynthesis and ECM receptor interaction pathways) and some harmful pathways (e.g., African trypanosomiasis, Chagas disease) were upregulated, whereas some other beneficial pathways (e.g., flavone and flavonol biosynthesis) and harmful pathways (e.g., cytochrome P450) were downregulated (Fig. S5B). This result, the overall redistribution of beneficial and harmful genera without affecting oocyte quality, is consistent with the previous proposal that AOS treatment shapes a new flora environment to maintain host metabolic homeostasis and oocyte quality (43).

**Fig 7:**
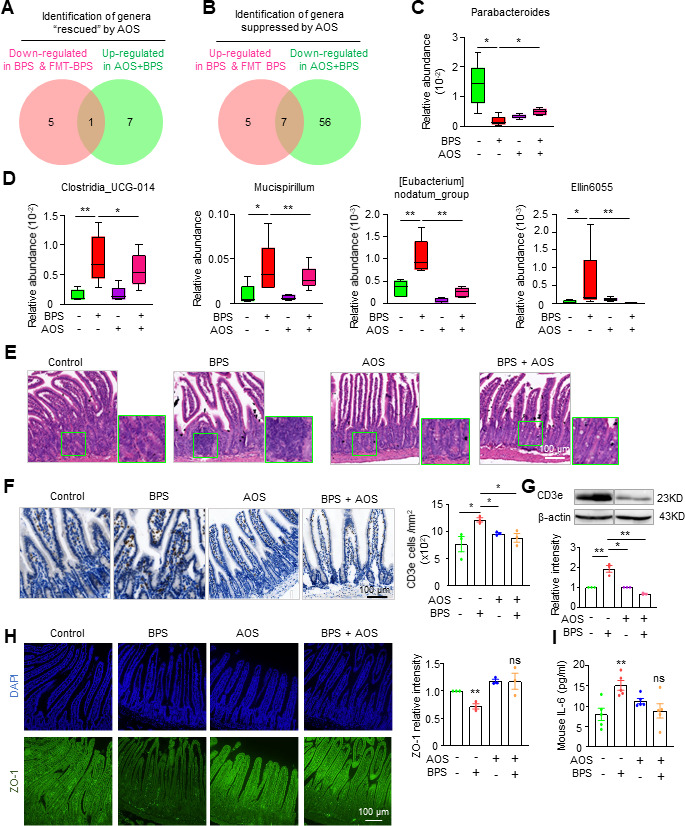
AOS prevents/rescues BPS-induced gut microbiota dysbiosis and inflammation. (**A**) Identification of bacteria “rescued” by AOS. (**B**) Identification of bacteria suppressed by AOS. (**C**) Representative genera “rescued” by AOS. (**D**) Representative genera suppressed by AOS. (**E**) H&E staining of intestinal sections to show that AOS in combination with BPS prevents intestinal lymphocyte infiltration that occurred in BPS-only treated mice. Scale bar, 100 µm. (**F**) Immunostaining of CD3e in intestinal sections and quantification of CD3e cell density. Scale bar, 100 µm. (**G**) Western blot and quantification of CD3e protein abundance in the intestine. (**H**) Immunostaining of ZO-1 (green) in intestinal sections and quantification of the relative intensity of ZO-1. DNA was counterstained with DAPI (blue). Scale bar = 100 µm. Representative images of control and BPS were duplicated from corresponding images in Fig. 4E. (**I**) Quantification of interleukin-6 in blood by ELISA. Error bars, SEM from 5 (**C, D, I**) and 3 (**E, F, G, and H**) independent experiments. one mouse per treatment in each experiment. n.s., *P* ≥ 0.05, *, *P* < 0.05, **, *P* < 0.01 by Wilcoxon rank-sum test (**C and D**) or Student’s *t*-test (**F–I**). Different treatments (control, BPS, FMT-control, FMT-BPS, AOS, and AOS/BPS) were performed in the same group of experiments. Data for control and BPS (**F–I**) were taken from the corresponding data in Fig. 4 (C–F).

If BPS acts (largely) through the gut microbiota, BPS-treated and FMT-BPS-treated mice would show similar changes in beneficial/harmful bacteria, and if AOS prevents or recues BPS-induced damage via the gut microbiota, AOS/BPS treatment would increase the beneficial genera and decrease the harmful genera compared with BPS treatment. Theoretically, the beneficial bacterial genera would be those upregulated in AOS/BPS-treated mice and downregulated in both BPS-treated and FMT-BPS-treated mice (Fig. 7A). Similarly, the harmful bacterial genera would be those upregulated in BPS-treated and FMT-BPS-treated mice, but downregulated in AOS/BPS-treated mice (Fig. 7B). Following this logic, *Parabacteroides* was identified as the possible beneficial genus to prevent BPS-induced impairment (Fig. 7A and C; Table S3). Indeed, *Parabacteroides* has already been reported to function in anti-inflammation (44), and thus, this result also validated our analysis strategy. Accordingly, seven genera were identified as possible harmful bacteria induced by BPS (Fig. 7B; Table S3). Among them, at least four genera (*Clostridia_UCG-014*, *Mucispirillum*, *[Eubacterium] nodatum_group*, and *Ellin6055*) have important functions in the inflammation development (Fig. 7D [21]). Therefore, AOS prevents or rescues BPS-induced damage to oocytes by increasing beneficial and suppressing harmful gut bacteria to create a new flora environment. We also noted that more harmful bacterial genera were identified compared with beneficial genera (seven vs one genera; Fig. 7A and B; Table S3). A similar phenomenon has also been observed when AOS is used to prevent/rescue intestinal inflammation (45). These observations seem to indicate that the main role of AOS is to suppress the generation of harmful bacteria, especially in the presence of toxins.

AOS can improve the intestinal microbiota and thus the metabolic homeostasis to prevent inflammation (43). Our further investigation showed that AOS alone or AOS/BPS treatment did not appear to cause the small intestinal lymphocyte infiltration or intestinal inflammation (Fig. 7E through I). This result further supports the idea that AOS improves the intestinal microenvironment to prevent BPS-induced damage to the intestine.

**Fig 8:**
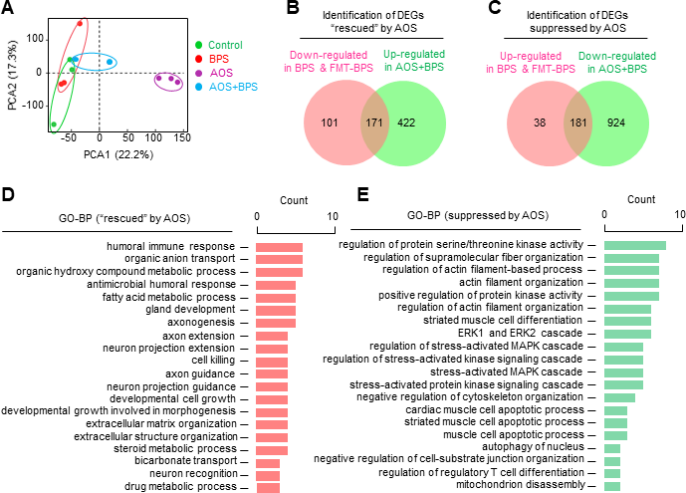
AOS prevents/rescues BPS-induced deleterious effects on oocyte transcriptional profile. (**A**) PCA analysis of oocyte RNA-seq data from different experimental groups. *n* = 3 independent experiments. one mouse per treatment in each experiment. (**B**) Identification of oocyte DEGs “rescued” by AOS. (**C**) Identification of oocyte DEGs suppressed by AOS. (**D**) GO enrichment analysis of oocyte DEGs from panel **B**. (**E**) GO enrichment analysis of oocyte DEGs from panel **C**.

RNA-seq analysis of oocytes from mice treated with AOS and BPS/AOS was performed to further explore the role of AOS. PCA analysis of the RNA-seq data showed that AOS treatment was well separated from other treatments (Fig. 8A). GO-BP analysis of DEGs from AOS treatment showed that protein synthesis and secretion, hormone transport/secretion, and glucose metabolism were significantly upregulated. However, inflammation-related signaling pathways were downregulated (Fig. S6). These results indicate a potential role for AOS in anti-inflammation and enhancement of the oocyte development by promoting protein/hormone synthesis and energy metabolism.

Following the same strategy as for the differential bacterial analysis, beneficial biological processes would be associated with DEGs “rescued” by AOS, that is, the expression of genes is downregulated in oocytes from BPS-treated and FMT-BPS-treated mice, whereas it is upregulated in oocytes from AOS/BPS-treated (compared with BPS-treated) mice (Fig. 8A and B). Harmful biological processes would be associated with DEGs suppressed by AOS, that is, the expression of genes is upregulated in oocytes from BPS-treated and FMT-BPS-treated mice, whereas it is downregulated in oocytes from AOS/BPS-treated (compared with BPS-treated) mice (Fig. 8A and C). GO-BP analysis revealed that 171 DEGs rescued by AOS were mainly enriched in extracellular matrix/structure, cell growth, immune response, transport, and steroid metabolism processes (Fig. 8D). Among them (Fig. S7A through E), many genes, such as semaphorin *Sema3a*, the zinc transport protein *Slc39a8, Dvl1*, *Cftr*, and transgenic Notch reporter (*Tnr*), have important functions in promoting oocyte development and maturation (46, 47).

GO-BP analysis also revealed that 181 DEGs suppressed by AOS were mainly enriched in processes such as actin/cytoskeleton organization, ERK cascade, stress-activated protein kinase (including MAPK) signaling cascade, apoptosis, mitochondrion disassembly, etc (Fig. 8E). Downregulation of these genes could protect oocytes from oxidative stress and cell death (Fig. S7F through J). For example, autophagy-related genes *Atg9b* and *Atg3* induce mitochondrial pathway-mediated cell death (48); *Arhgap6* inhibits cell proliferation, migration, and invasion, induces cell cycle arrest at the G0/G1 transition, and promotes cell apoptosis (49); *Dusp1* can suppress ERK signaling pathway and regulate mitochondrial MMP and ROS (30); and nicotinamide adenine dinucleotide phosphate (NADPH) oxidase 4 (*Nox4*) is a member of NADPH NOXs family, which is the main source of ROS generation (33). Therefore, downregulation of these genes could protect oocytes.

Our analyses of gut microbiota and oocyte transcriptomes from AOS- and AOS/BPS-treated mice suggest that AOS prevents BPS-induced intesinte inflammation and maintains oocyte quality by reshaping the gut flora.

## DISCUSSION

We have shown that BPS exposure impairs oocyte quality by inducing dysbiosis of gut microbiota, resulting in increased intenstinal permeability, inflammation, and immune response. Importantly, AOS improves the gut microenvironment, specifically reshaping the gut microbiota to prevent BPS-induced damage to the gut and oocytes.

Bisphenols can bind directly to the membrane and nuclear estrogen receptors and also to other receptors (e.g., estrogen-related receptors and thyroid hormone receptors) to interfere with the expression of target genes, associated signaling pathways, hormone synthesis and metabolism, and the feedback control system (e.g., hypothalamic-pituitary-gonadal/adrenal/thyroid/ovarian axis) (50). Bisphenols-induced oxidative stress (probably consequently inflammation and immune response) has also been frequently observed, which would lead to mitochondrial dysfunction, cell apoptosis, and autophagy and ultimately result in many health problems. Oxidative stress therefore appears to be another major toxic mechanism for bisphenols (51). Consistently, antioxidants can protect against bisphenol-induced male and female reproductive disorders (51, 52).

BPS is primarily ingested through the mouth, and the gut is the first target organ. Many studies have shown that BPA can induce dysbiosis of the gut microbiota, impair oocyte quality, damage the intestinal barrier, and increase inflammation (16). Our current study showed that BPS has similar adverse effects as its analog BPA. However, the relationship between the gut microbiota dysbiosis and other defects, especially the poor oocyte quality induced by bisphenols, is unclear. BPA and BPS can disrupt granulosa cell steroidogenesis, which affects oocyte development and alters oocyte quality (13). It is possible that BPS exposure induces gut microbiota dysbiosis, which further disrupts granulosa cell steroidogenesis and ultimately affects oocyte quality. Our further investigation with FMT provided the first strong evidence to support that BPS exposure first disrupts the gut microbiota, which then impairs oocytes. This idea is further supported by the finding that AOS can prevent BPS-induced adverse effects on oocytes. Further mechanistic evidence was obtained from oocyte RNA-seq and gut 16S rDNA sequencing, as discussed below.

Our 16S rDNA sequencing revealed that BPS and FMT-BPS induce a dysbiosis of the gut microbiota, specifically more harmful bacteria (including *Clostridia_UCG-014, [Eubacterium] nodatum_group, Family XIII AD3011 group, Rikenellaceae RC9 gut group,* and *Mucispirillum*) and fewer beneficial bacteria (including *Odoribacter*, *Parabacteroides*, and *Faecalibaculum*). These alterations are very similar to BPA-induced alterations as previously reported (16). These identified harmful bacteria with increased abundance can induce or be associated with intestinal permeability, inflammation, and oxidative stress by producing pro-inflammatory metabolites or disrupting associated metabolic processes (24). Additionally, the *[Eubacterium] nodatum_group* is also associated with preterm birth, miscarriage, and recurrent pregnancy loss, and the *Family XIII AD3011 group* is considered a biomarker for polycystic ovary syndrome (24). The identified beneficial bacteria with reduced abundance can produce short-chain fatty acids to reduce levels of pro-inflammatory factors and suppress inflammation (27). Therefore, the changes in the gut microbiota are thought to be associated with increased gut permeability and inflammation. RNA-seq of oocytes revealed that the stress-activated MAPK signaling pathway is upregulated to promote apoptosis and inflammatory responses and suppress the ERK signaling pathway required for follicle development. Overall, our current findings demonstrate that BPS-induced dysbiosis of the gut microbiota promotes intestinal inflammation and ultimately leads to oxidative stress and oocyte impairment.

AOS, as a product of alginate, shows many biological activities including antioxidant, immunoregulatory, anti-inflammatory, and anti-obesity (53). Due to the lack of β-1,4-glycosidic bond hydrolyzing enzyme, dietary AOS cannot be digested in the gastrointestinal tract of animals and humans but can be metabolized by microbiota in the cecum and colon, thereby modulating intestinal flora (54). AOS can rescue/prevent BPS-induced intestinal lymphocyte infiltration and instestinal inflammation. Further studies show that AOS reshapes the intestinal flora microenvironment induced by BPS: (i) increases the abundance of the beneficial bacteria, *Parabacteroides*, which play important roles in protecting the intestinal barrier and functioning in anti-inflammation (55); (ii) decreases the abundance of BPS-induced harmful bacteria, that is, *Clostridia_UCG-014, Mucispirllum, [Eubacterium] nodatum_group*, and *Ellin6055*), which play important roles in the development of inflammation. Therefore, AOS recues/prevents BPS-induced adverse effects via reshaping intestinal flora to maintain metabolic homeostasis to modulate the immunone response and protect the intestinal barrier function.

Finally, AOS rescues/prevents BPS-induced reduced oocyte quality. RNA-seq shows that AOS treatment was distinct from BPS/AOS treatments by PCA analysis. This is not surprising. The gut microbiota reshaped by AOS could be diferent, depending on the different microenvironments in the host (i.e., healthy or sick). This would be involved in the complicated interactions between host, microbiota, and metabolites, ultimately establishing new flora and metabolic homeostasis.

GO-BP analysis of DEGs showed that protein synthesis and secretion, hormone transport/secretion, and glucose metabolism were significantly upregulated in oocytes from AOS-treated mice compared with the control. However, inflammation-related signaling pathways were downregulated, suggesting that AOS may function in anti-inflammation and improve oocyte development by promoting protein/hormone synthesis and energy metabolism. Interestingly, GO-BP analysis of DEGs rescued by the AOS in BPS/AOS treatment is mainly enriched in extracellular matrix/structure, cell growth, immune response, transport, and steroid metabolism processes (46, 47). The supressed DEGs are mainly enriched in processes such as actin/cytoskeleton organization, ERK cascade, stress-activated protein kinase (including MAPK) signaling cascade, apoptosis, mitochondrion disassembly, etc. Downregulation of these genes could protect oocytes.

Our study also suggests that AOS reshapes the intestinal flora to improve intestinal homeostasis, thus preventing BPS-induced adverse changes in the intestine and oocytes. However, how AOS rescue oocyte quality is not clear. There is the possibility that AOS or its metabolite is transported to the ovaries and thus plays a more direct role in preventing oxidative stress and inflammation damage in oocytes. Interestingly, PCA analysis of RNA-seq shows that the AOS group is well separated from the others, whereas AOS/BPS, BPS, and the control groups do not appear to be clearly distinct. It is possible that the rescue of oocyte quality by AOS does not occur via direct effects on the oocyte transcriptome, but perhaps via indirect effects on other ovarian cells (i.e., immune cells or somatic cells) or on the neuroendocrine axis. Another less likely, but not excluded, possibility is that AOS may interact directly with BPS, thus suppressing/preventing its activity.

It appears that many (if not all) bisphenols-induced disorders are caused by metabolic, inflammatory, and immune dysregulation and, more importantly, are closely linked to bisphenols-induced alterations in gut microbiota (16). Our findings thus raise the important and intriguing question of whether other adverse effects of bisphenols are also caused by the gut microbiota dysbiosis, and if so, whether they can also be prevented or alleviated by AOS/antioxidant supplements. Further investigation is needed to answer these questions and develop preventive and therapeutic strategies.

## MATERIALS AND METHODS

### Animals

The 4-week-old female ICR mice were purchased from Beijing Vital River Co. Ltd. Totally, ~200 mice were used in this study. The animals were maintained in a mass air-displacement room with a 12 h light-dark cycle at 22°C ± 2°C and a relative humidity of 50% ± 10%. Food and drinking water were provided to the animals *ad libitum*. Foods containing ∼20% protein, ∼12% fat, multiple vitamins, and minerals were purchased from Xie-tong Biomedicine (SFS9112).

### BPS and AOS treatment

BPS was purchased from Sigma-Aldrich (Cat#103039-100G, Sigma). Based on the BPS in environmental sources or in human urine, the median of estimated daily exposure to BPS can be up to ~1.7 µg/day for the general population and ~22 µg/day for the occupationally exposed individuals (4). For an individual with a body weight (bw) of 60 kg, the daily exposure dose would be ~30 ng/kg bw/day for the general population and ~370 ng/kg bw/day for the occupationally exposed individuals, respectively. To provide information on potential health hazards, the 28-day repeated dose oral toxicity study is widely used in rodents (56).

In this study, 4-week-old female mice (~20 g) were given 1 mg/kg bw/day BPS by gavage daily for 4 weeks, and the total BPS is 560 µg (1 mg/kg bw/day × 20 g × 28 days). Five hundred and sixty µg corresponds to ~90 years and ~7 years for the general exposure population and occupational exposure population, respectively. In addition, the natural environmental exposure and occupational exposure doses may increase with more BPS substitutes for BPA.

Alginate oligosaccharides (AOS) were purchased from Bozhihuili Co. Ltd (Cat#9012-76-4). AOS at 10 mg/kg bw/day can effectively mitigate intestinal mucositis induced by Busuflfan (an anticancer drug) (43); hence, we used 10 mg/kg bw/day as a rescue dose of AOS. Four-week-old female ICR mice were given 100 µL corn oil (C116025; Aladdin, China), 1 mg/kg bw BPS, 10 mg/kg bw AOS, or 1 mg/kg bw BPS and 10 mg/kg bw AOS by oral gavage daily for 4 weeks. Mice were weighed once a week to correct for BPS and AOS doses.

### Fecal microbiota transplantation

Fecal samples were collected from the small intestine of control or 1 mg/kg BPS-treated mice, mixed with 20% sterile glycerol to a final concentration of 0.05 g/mL, and filtered through 70 µm cell strainer and frozen at −80 ^o^C (15). Recipient mice were fasted for 6 h and orally gavaged with 200 µL Abx (1 g/L ampicillin, 0.5 g/L neomycin, 0.5 g/L vancomycin, and 1 g/L metronidazole) for 3 days (57). These mice were then randomly divided into two groups and were given 200 µL fecal suspension obtained from the control and BPS-treated mice, respectively, once daily for 4 weeks.

### Oocyte collection

After 4 weeks of treatment, these 8-week-old female ICR mice were sacrificed. Fully grown germinal vesicle (GV) oocytes were isolated from antral follicles and transferred to M2 medium (M7167, Sigma-Aldrich, America) supplemented with 2.5 µM milrinone. After removal of the granule cells surrounding oocytes, the oocytes were washed, transferred to pre-equilibrium M16 medium covered with mineral oil, and cultured at 37°C in a humidified atmosphere containing 5% CO_2_. The first polar body extrusion was examined after 12 h.

### *In vitro* fertilization

After treatment, 8 week ICR female mice were injected with 8 IU pregnant mare serum gonadotrophin (PMSG) (Ningbo Second Hormone Factory, China). After 48 h, mice were injected with 8 IU human chorionic gonadotropin (hCG) (Ningbo Second Hormone Factory, China). After a further 16 h, the cumulus-oocyte complex (COC) in the oviductal ampulla was collected in HTF medium (MR-070, Millipore, United States) and incubated with capacitated sperm at 37°C under a humidified atmosphere containing 5% CO_2_. After 6 h, fertilized oocytes (with two pronuclei) were examined. All oocytes were transferred into a pre-balanced KSOM medium (MR-121, Millipore, United States) at 37°C in a CO_2_ incubator and two-cell embryos, four-cell embryos, and blastocysts were examined at 18 h, 24 h, and 72 h, respectively.

### Annexin-V staining

Annexin V-FITC Apoptosis Kit (C1062S, Beyotime, China) was used to detect early apoptosis of mouse oocytes. To remove the zona pellucida, the oocytes were incubated in acid Tyrode’s solution and observed under a stereomicroscope. When no zona pellucida structure was seen, the oocytes were immediately transferred to M2 medium. The oocytes were then transferred to 30 µL annexin V-FITC working solution (1:40) and incubated at 25 ^o^C in the dark for 15 min. After washing with PBS solution, oocytes were mounted on a glass-bottom dish and observed under a rotary laser confocal microscope (Dragonfly, Andor Technology). Early apoptotic oocytes show green fluorescence on the cytoplasmic membrane.

### Mitochondrial membrane potential assay

MMP was examined by JC-1 assay using the Mitochondrial membrane potential assay kit (C2006, Beyotime, China) according to the manufacturer’s instructions. Briefly, GV oocytes were incubated in the JC-1 working solution (50 µL JC-1 stock solution, 8mL M2 medium, and 2 mL JC-1 staining buffer) covered with mineral oil in the dark for 20 min in a CO_2_ incubator at 37 ^o^C. After washing with 1× PBS solution, the samples were examined under a fluorescence microscope (IX71, Olympus), and red and green fluorescence images were acquired using the same imaging parameters. MMP was calculated as the ratio of red-to-green fluorescence intensity.

### ATP content detection

ATP levels were determined using an Enhanced ATP Assay Kit (S0027, Beyotime, China). For each reaction, 10 GV oocytes were pooled and lysed by rapid freeze-thaw cycles, enzyme working solution was added and incubated at room temperature for 3–5min, the mixture was transferred to an opaque 96-well plate, and the luminescence was examined using a luminometer (EnSpire, PerkinElmer, 0.01 p.m. sensitivity). The ATP level was then determined by comparing the luminescence intensity with a five-point standard curve (0.02, 0.04, 0.06, 0.08, and 0.1 nM).

### mRNA-sequencing and data analysis

mRNA seq was performed with ~20 oocytes in each experiment by Smart-seq2. Reverse transcription and amplification were performed according to the standard protocol of 10K genomics, Shanghai China. The Illumina NovaSeq 6000 sequencing platform was used (Illumina, San Diego, CA), and the PE150 sequencing mode was adopted.

Raw data were aligned to the genome of mm39 by Hisat2 (2.1.0). Feature count (v2.0.1) was used to calculate the number of genes. Different genes were determined by limma with the standard threshold of “padj <0.05 and |log2FoldChange| > 1.” GO pathway analysis was performed using ClusterProfiler (v4.0.1) (58). Different GO terms were defined by the *P*-value < 0.05. The expression level of mRNA was normalized by fragments per kilobase of the exon model per million mapped fragments (FPKM).

### DNA extraction and 16S rDNA sequencing

After treatment, 8-week female ICR mice were sacrificed to collect the luminal contents of the small intestine. Genomic DNA was extracted using 2× CTAB (cetyltrimethylammonium bromide) (LS00066, Solarbio Science & Technology, China) with 1% mercaptoethanol and DNA extraction reagent (enol:chloroform:isoamylol = 25:24:1) (P1021, Solarbio Science & Technology, China). DNA concentration was determined using the NanoDrop ND-2000 (Thermo Fisher Scientific). The 16S rRNA genes of the V4 regions were amplified using a barcode-specific primer pair (515F: GTGCCAGCMGCCGCGGTAA; 806R: GGACTACHVGGGTWTCTAAT). The library was sequenced on an Illumina NovaSeq platform, and 250 bp paired-end reads were generated.

The 16S rDNA sequence result was analyzed using the Easy Amplicon pipeline (59). Paired-end reads were merged using FLASH (VI.2.7). Clean amplicons were denoised into amplicon sequence variants (ASVs) in *de novo* mode by Vsearch-unoise3 (60) for further analysis. Taxonomy was assigned to all ASVs using the RDP classifier within QIIME2 and the Silva reference data set (61). Alpha diversity (observed features, Shannon) and beta diversity (Bray curtis distances, principal coordinate analysis [PCoA]) were also analyzed using QIIME2. Linear discriminant analysis (LDA) and effect size (LEfSe) (http://www.ehbio.com/ImageGP/index.php/Home/Index/LEFSe.html) were used to determine the differential bacteria. PICRUSt2 was used to predict the KEGG pathways of the bacteria (62).

### ELISA

Serum IL-6 levels were determined using an IL-6 ELISA Kit (431304, BioLegend) according to the manufacturer’s instructions. Briefly, non-specific binding was blocked for all wells of a 96-well plate, and samples were added to the wells, sealed, and incubated for 2 h at room temperature with shaking. The plate was washed; diluted detection antibody solution was added and incubated for 1 h at room temperature with shaking. After washing, diluted Avidin-HRP solution was added and incubated for 30 min with shaking. The plate was washed again, freshly mixed TMB substrate was added, and incubated for 20 min in the dark. Stop solution was then added to stop the reaction. The absorbance at 450 nm and 570 nm was read immediately. The absorbance at 570 nm was subtracted from the absorbance at 450 nm and compared with the standard curve.

### Histological analysis

Small intestine tissues were fixed in 4% paraformaldehyde, embedded in paraffin, and sectioned (5 µm thick). They were stained with haematoxylin and eosin (H&E) for histopathological analysis or subjected to antigen retrieval and immunostaining for inflammatory analysis.

### Quantitative reverse transcription PCR

Total RNA from ooctyes was extracted using RNeasy Mini Kit (74104, QIAGEN, Germany) according to the manufacturer’s protocol. HiScript II Q RT SuperMix (Q111, Vazyme Biotech, China) was used to remove the residual genomic DNA and synthesize the cDNA. PCR was performed in a 10 µL reaction system (1 µL cDNA template; 5 µL SYBR Green Mix (RK21203, ABclonal); 0.25 µL 10 µM forward primer; and 0.25 µL 10 µM reverse primer; 3.5 µL ddH2O) with the following amplification conditions: pre-denaturation at 95°C for 3 min, 45 cycles of denaturation at 95°C for 5 s, and annealing at 60°C for 34 s. All reactions were performed in triplicate. Primers are listed in Table S4.

### Detection of BPS in fecal suspension

BPS in fecal suspension was detected by LC/MS. Briefly, 100 µL fecal suspension was mixed with 300 µL methanol and centrifuged, and the supernatant was transferred to a clean tube and air dried. In total, 100 µL of 50% methanol was added to the sample, and 5 µL of the supernatant was used for detection with an Agilent 1290 Infinity II triple quadrupole mass spectrometer (American). The UPLC conditions were as follows: 0–3.5 min, 20%–100% B; 3.5–4.5 min, 100%, 100% B; 4.5–7 min, and 100%–20% B. Multiple reaction monitoring (MRM) in negative ionization mode was used: *m*/*z* 249.1 → 108.0 for BPS (collision energy 26 eV). The concentration of BPS was determined by comparison with the standard curve.

### Antibodies

Primary antibodies used were anti-CD3 rabbit pAb (WB and ICC, 1:1,000, Servicebio, #GB11014), mouse anti-β-ACTIN (WB, 1:3,000; Proteintech, #66009-1-Ig), and ZO-1 antibody (ZO1-1A12) (IF, 1:200, Invitrogen, #33-9100).

Secondary antibodies were HRP-conjugated goat anti-rabbit IgG (WB, 1:5,000; Proteintech, #SA00001-2), HRP-conjugated goat anti-mouse IgG (WB, 1:5,000; Proteintech, #SA00001-1), CoraLite488-conjugated goat anti-mouse IgG (IF, 1:300; Proteintech, SA00013-1), and HRP-conjugated goat anti-rabbit IgG (ICC, 1:200, Servicebio, GB23303).

### Statistical analysis

Data were presented as mean ± SEM or SD. The statistical significance of the differences was determined by Student’s *t*-test or Wilcoxon rank-sum and indicated as follows: n.s., not significant, *P* ≥ 0.05; *, *P* < 0.05; **, *P* < 0.01; ***, *P* < 0.001.

## Data Availability

RNA-seq data are available at NCBI (SRA Bioproject, accession number PRJNA1045813). The 16S rDNA sequencing data are available at NCBI (SRA Bioproject, accession number PRJNA1045335.

## SUPPLEMENTAL MATERIAL

Supplemental material msystems.00912-24-s0001.pdf Figures S1-S7 and Tables S1-S5.

ASM does not own the copyrights to Supplemental Material that may be linked to, or accessed through, an article. The authors have granted ASM a non-exclusive, world-wide license to publish the Supplemental Material files. Please contact the corresponding author directly for reuse.
