# Supplementary material for: Bisphenol S impairs oocyte quality by inducing gut microbiota dysbiosis
Source: mSystems. 2024 Dec 20;10(1):e00912-24. doi: 10.1128/msystems.00912-24 (PMC11748550; doi:10.1128/msystems.00912-24)
Supplement: Supplemental material — Figures S1-S7 and Tables S1-S5. [file msystems.00912-24-s0001.pdf]

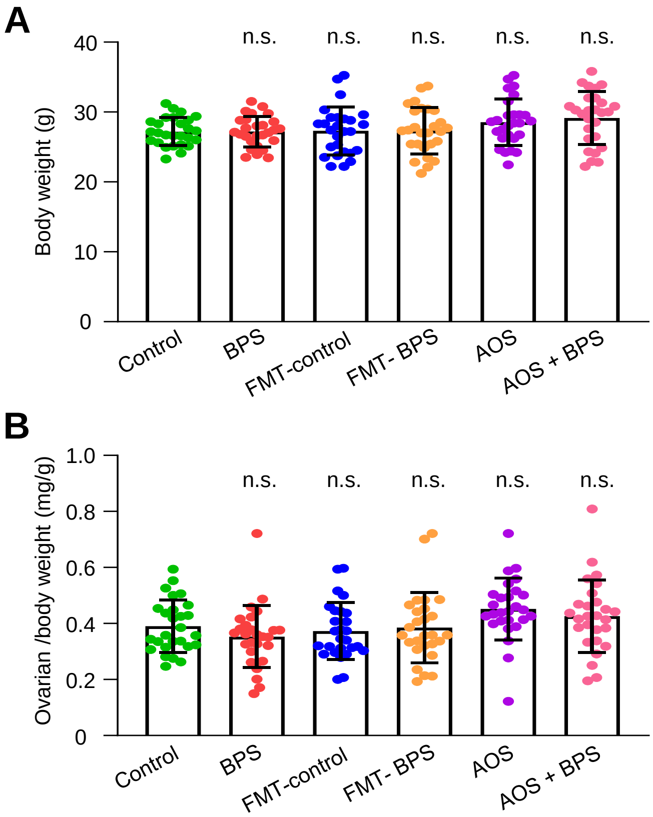

Figure S1. Ovarian and body weight of mice were not affected after different treatments.

(A) Quantification of body weight with different treatments. **(B)** Quantification of the ratio of ovarian weight to body weight (mg/g). Error bars, SD. 27 mice were used in each group. n.s., $p \geq 0.05$; Student’s t-test.

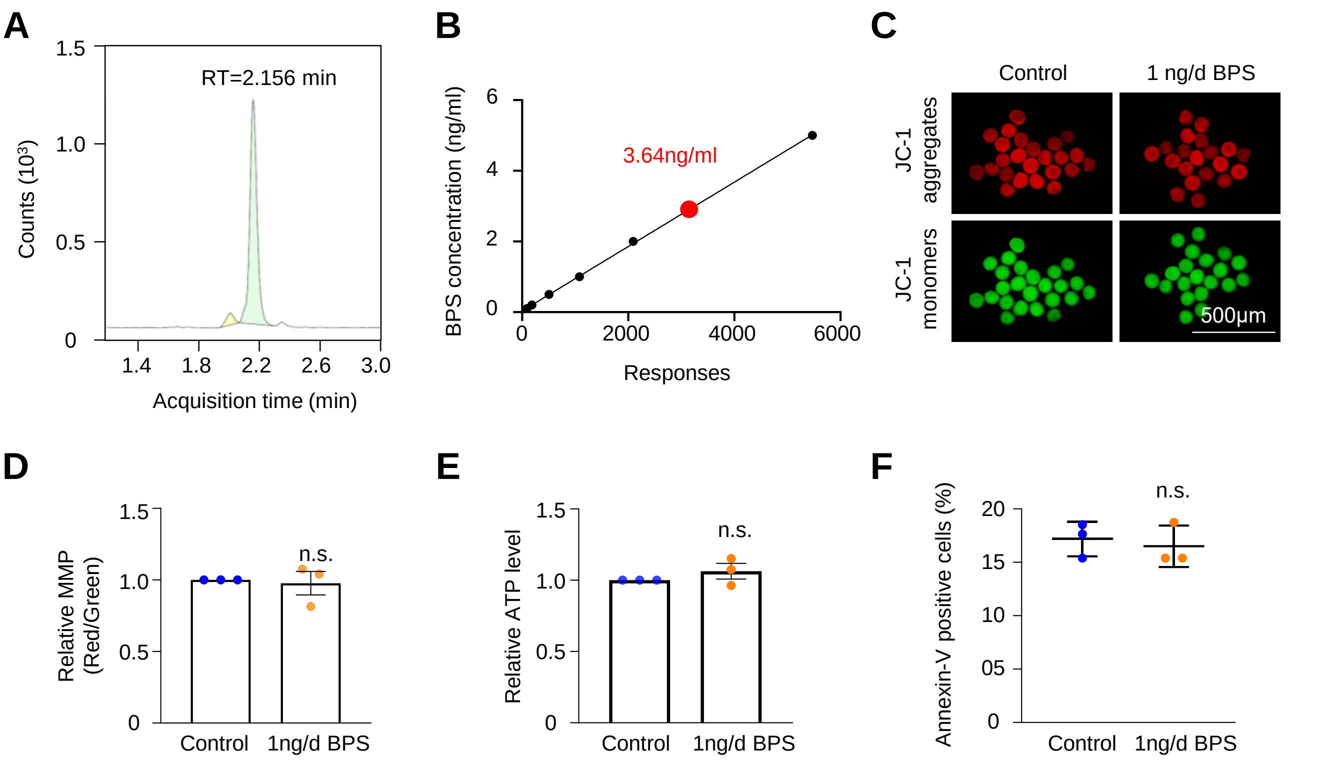

Figure S2. Residual BPS in feces does not affect oocyte quality.

(A) LC/MS detection of residual BPS in feces. The area enclosed by the curve and the baseline represents the relative content of BPS. **(B)** Comparion with the standard curve to determine the concentration of residual BPS in feces (the red dot, 3.64 ng/ml). **(C, D)** Representative images (C) and quantification (D) of the JC-1 signal. The ratio of red to green signal intensity indicates the MMP levels. For FMT, recipient mice were treated by intra-gastric gavage with 200ul of fecal suspension daily for 28 consecutive days. The residual BPS in the feces is 3.64 ng/ml (B), so there is 0.73ng BPS in 200ul of fecal suspension. To explore whether this amount of BPS could affect oocytes, mice were treated with 1ng BPS per day (1ng/d) by intra-gastric gavage for 28 consecutive days. MMP, relative ATP levels, and apoptosis in oocytes were then examined. Error bars, SEM for 3 independent experiments. ~25 GV oocytes from two mice per treatment in each experiment. Totally, 70 and 77 GV oocytes for different treatments, respectively. n.s., $p \geq 0.05$; Student's t-test. **(E)** Relative ATP levels in oocytes. Error bars, SEM from 3 independent experiments. 10 GV oocytes from one mouse per treatment in each experiment. Totally, 30 GV oocytes for different treatments, respectively. n.s., $p \geq 0.05$; Student's t-test. **(F)** Quantification of oocytes with Annexin-V signal. Error bars, SEM from 3 independent experiments. ~25 GV oocytes from 1-2 mice per treatment in each experiment. Totally, 79 and 66 GV oocytes for different treatments, respectively. n.s., $p \geq 0.05$; Student's t-test.

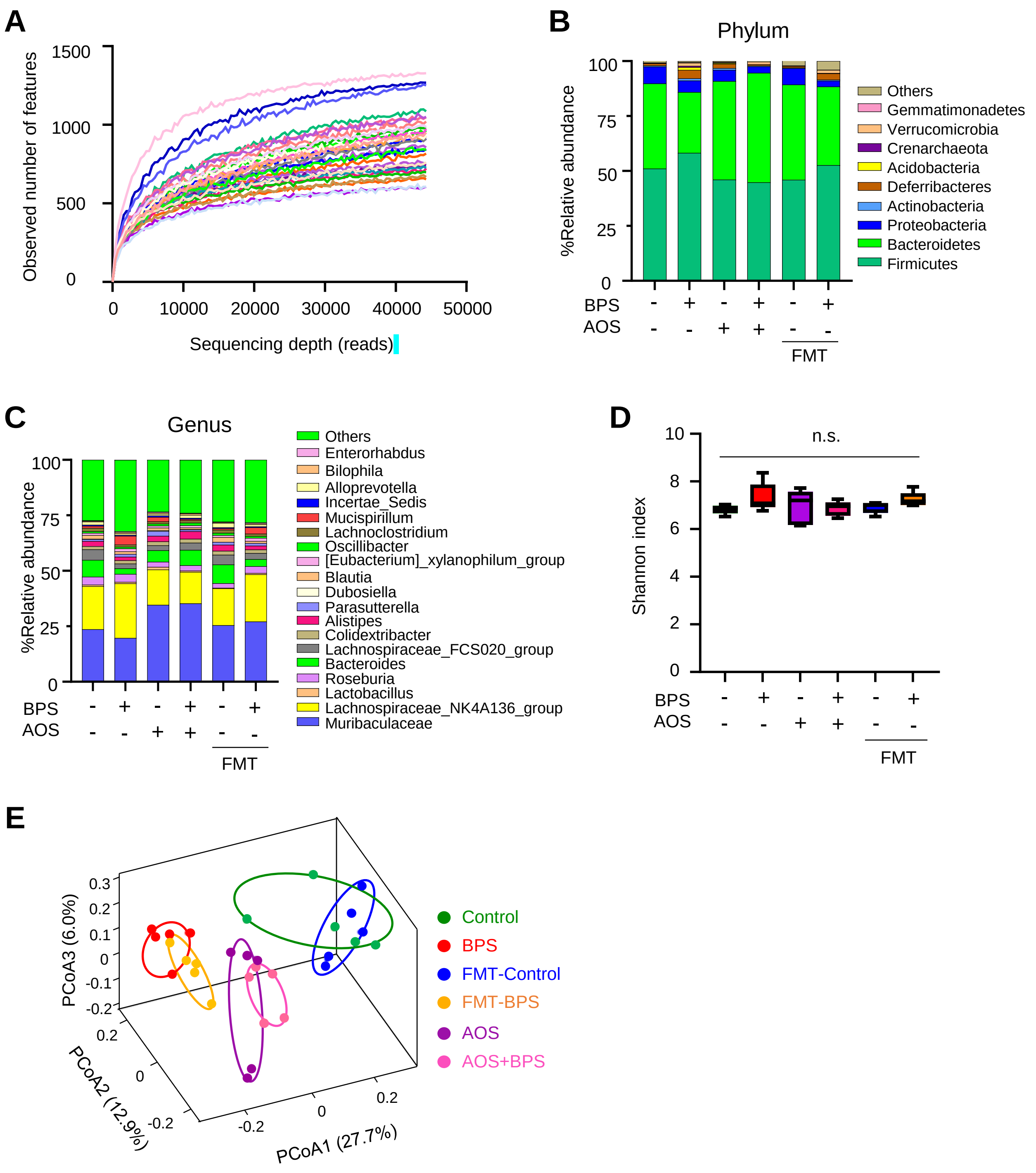

Figure S3. Composition and structure of the gut microbiome.

(A) Rarefaction curve based on the species diversity showed sufficient coverage of sequences. **(B, C)** Dominant phyla (B) and genera (C). **(D)** Shannon indices show no differences between different treatments. n.s., p≥0.05; Student's t-test. **(E)** PCoA analysis of 16S rDNA from different treatments. n=5 independent experiments. One mouse per treatment in each experiment.

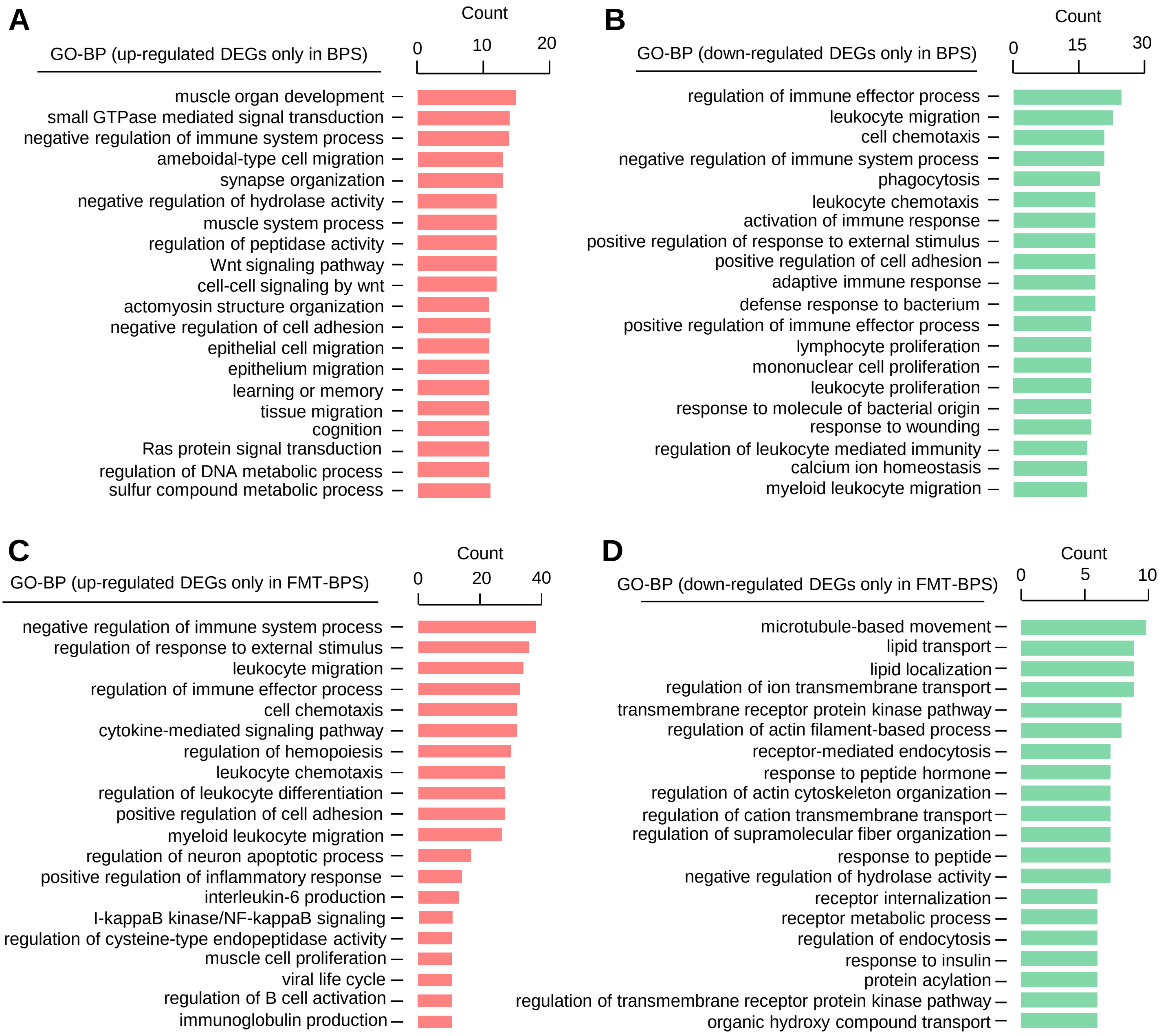

Figure S4 GO enrichment analysis of BPS or FMT-BPS specific DEGs.

(A) GO-BP analysis of up-regulated DEGs only in BPS but not FMT-BPS treated mouse oocytes. **(B)** GO-BP analysis of down-regulated DEGs only in BPS but not FMT-BPS treated mouse oocytes. **(C)** GO-BP analysis of up-regulated DEGs only in FMT-BPS but not BPS treated mouse oocytes. **(D)** GO-BP analysis of down-regulated DEGs only in FMT-BPS but not BPS treated mouse oocytes.

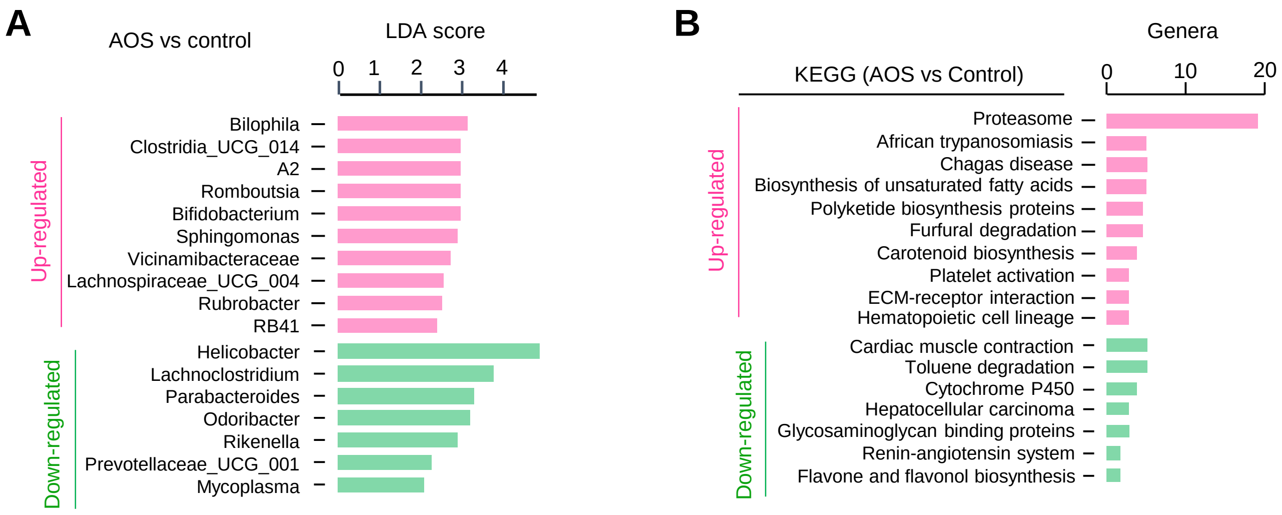

Figure S5. AOS improves gut microbiota.

(A) LEfSe analysis of gut microbiota from AOS-treated mice. **(B)** KEGG analysis of genera with altered abundance from (A).

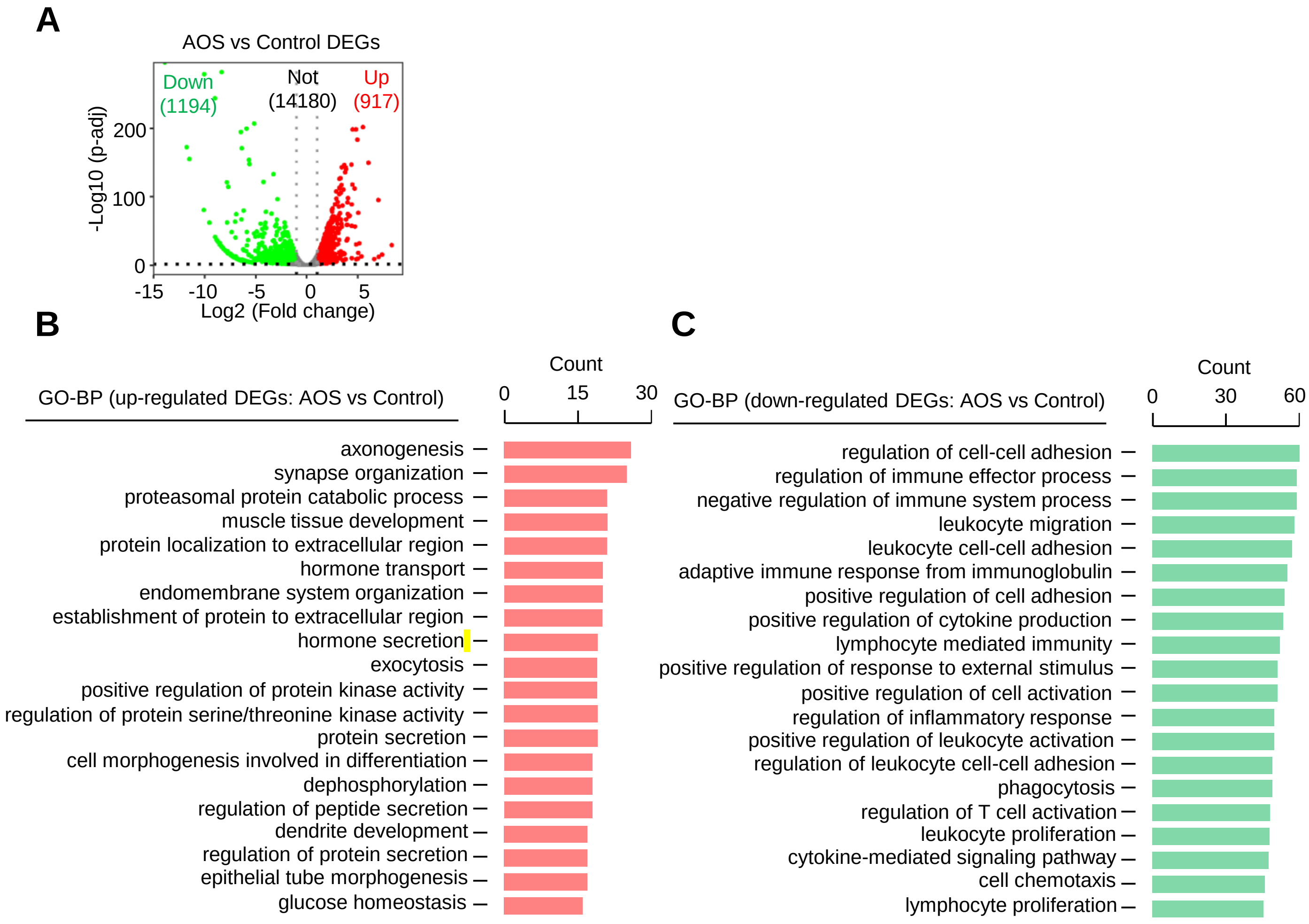

Figure S6. Analysis of oocyte RNA-seq data from AOS-treated mice.

(A) Volcano plots showing DEGs from AOS-treated mouse oocytes. **(B)** GO enrichment analysis of up-regulated DEGs from AOS-treated mouse oocytes. **(C)** GO enrichment analysis of down-regulated DEGs from AOS-treated mouse oocytes.

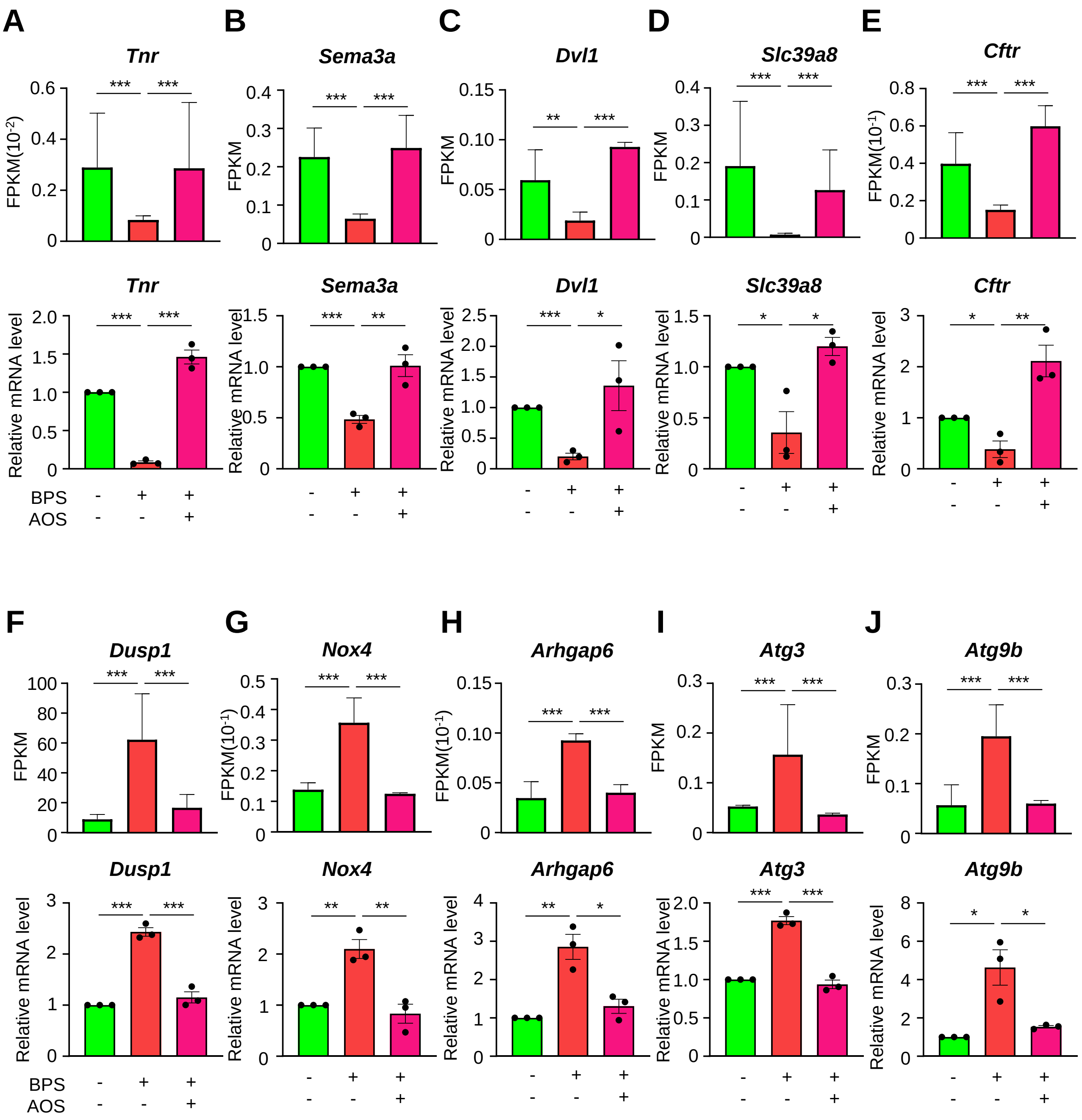

Figure S7. Representative DEGs from RNA-seq of AOS-treated oocytes and corresponding RT-qPCR verification. (A-E) Representative DEGs of “rescued” by AOS (top row) and RT-qPCR (bottom row). **(F-J)** Representative DEGs suppressed by AOS (top row) and RT-qPCR (bottom row). Error bars, mean ± SEM (n=3 RNA-seq except 2 were performed for AOS/BPS treated mice, or 3 RT-qPCR). one mouse per treatment in each experiment.*, $p<0.05$; **, $p<0.01$; ***, $p<0.001$. Significance analysis was performed using edgeR (RNA-seq) or Student’s t-test (RT-qPCR).

Table S1. Identified genera with altered abundance in BPS and FMT-BPS treated mice.

**Alteration**	**Genera**
Up-regulated in BPS only (29)	*Romboutsia; Clostridium_sensu_stricto_1; Bilophila; Sphingomonas; RB41; Legionella; Rubrobacter; Pontibacter; UCG_009; Microvirga; Acidibacter; Rhizobium; Azospirillum; Delftia; Caulobacter; Bryobacter; UBA1819; Nordella; Altererythrobacter; Flavisolibacter; Anaerocolumna; Blastococcus; Gastranaerophilales; Flavitalea; Variovorax; 67_14; Cellvibrio; AKAU4049; Ramlibacter*
Up-regulated in FMT-BPS only (13)	*Lachnospiraceae_NK4A136_group; Mucispirillum; Rikenellaceae_RC9_gut_group; Akkermansia; [Eubacterium] xylanophilum_group; Clostridia_UCG_014; GCA_900066575; Lactobacillus; RF39; Intestinimonas; UCG_009; [Eubacterium] ventriosum_group; [Eubacterium] nodatum_group; Lachnospiraceae_UCG_006; [Eubacterium] siraeum_group; MND1; NK4A214_group; Tyzzerella; Subgroup_10; Escherichia_Shigella; Ellin6055; Tuzzerella; KD4_96; Family_XIII_AD3011_group; Acinetobacter*
Up-regulated in both BPS and FMT-BPS (12)	*Mucispirillum; Rikenellaceae_RC9_gut_group; Clostridia_UCG_014; RF39; [Eubacterium] ventriosum_group; [Eubacterium] nodatum_group; [Eubacterium] siraeum_group; UCG_009; Tyzzerella; NK4A214_group; Ellin6055; Family_XIII_AD3011_group*
Down-regulated in BPS only (2)	*Lachnospiraceae_FCS020_group; Candidatus_Arthromitus;*
Down-regulated in FMT-BPS only (9)	*Bacteroides; Blautia; F082; Rikenellaceae; Mycoplasma; Coriobacteriaceae_UCG_002; Rs_E47_termite_group; Ileibacterium; Candidatus_Stoquefichus*
Down-regulated in both BPS and FMT-BPS (6)	*Parabacteroides; Odoribacter; Desulfovibrio; Rikenella; Faecalibaculum; Tritrichomonas*

Table S2. KEGG analysis of genera with altered abundance in BPS and FMT-BPS treated mice.

Alteration	Pathway	Genera
**Up-regulated**	• Transporters	*[Eubacterium]_nodatum_group; [Eubacterium]_siraeum_group; [Eubacterium]_ventriosum_group; Clostridia_UCG-014; Ellin6055; Family_XIII_AD3011_group; Mucispirillum; NK4A214_group; RF39; Rikenellaceae_RC9_gut_group; Tyzzerella; UCG-009*
	• Translation	*[Eubacterium]_nodatum_group; [Eubacterium]_siraeum_group; [Eubacterium]_ventriosum_group; Clostridia_UCG-014; Ellin6055; Family_XIII_AD3011_group; NK4A214_group; RF39; Rikenellaceae_RC9_gut_group; Tyzzerella; UCG-009*
	• Thyroid hormone synthesis	*[Eubacterium]_nodatum_group; [Eubacterium]_ventriosum_group; Clostridia_UCG-014; Ellin6055; Family_XIII_AD3011_group; RF39; Rikenellaceae_RC9_gut_group*
	• Geraniol degradation	*Clostridia_UCG-014;Ellin6055;Rikenellaceae_RC9_gut_group;UCG-009*
	• Carotenoid biosynthesis	*Ellin6055;RF39;Rikenellaceae_RC9_gut_group*
	• D-Arginine and D-ornithine metabolism	*[Eubacterium]_nodatum_group; Clostridia_UCG-014;Family_XIII_AD3011_group*
	• Ethylbenzene degradation	*Ellin6055;Rikenellaceae_RC9_gut_group;UCG-009*
	• Staphylococcus aureus infection	*[Eubacterium]_nodatum_group;Anaerovoracaceae;Clostridia_UCG-014;Family_XIII_AD3011_group*
	• African trypanosomiasis	*Ellin6055; Rikenellaceae_RC9_gut_group; NK4A214_group*
	• Amphetamine addiction	*Clostridia_UCG-014;Ellin6055*
	• Chagas disease	*Ellin6055; Rikenellaceae_RC9_gut_group*
	• Cocaine addiction	*Clostridia_UCG-014;Ellin6055*
	• Dopaminergic synapse	*Clostridia_UCG-014;Ellin6055*
	• Serotonergic synapse	*Clostridia_UCG-014;Ellin6055*
	• Polycyclic aromatic hydrocarbon degradation	*Ellin6055*
	• Sesquiterpenoid and triterpenoid biosynthesis	*Rikenellaceae_RC9_gut_group*
	• Steroid biosynthesis	*Rikenellaceae_RC9_gut_group*
	• RIG-I-like receptor signaling pathway	*Clostridia_UCG-014*
	• Retrograde endocannabinoid signaling	*Mucispirillum*
**Down-regulated**	• Ether lipid metabolism	*Odoribacter; Parabacteroides*
	• Linoleic acid metabolism	*Odoribacter; Parabacteroides*
	• Steroid hormone biosynthesis	*Parabacteroides*

Table S3. Identified genera with altered abundance in BPS and FMT-BPS treated mice.

Alteration	Genera
Up-regulated in AOS + BPS vs BPS (7)	*Muribaculaceae; Bacteroides; Lachnospiraceae_UCG_001; Prevotellaceae_UCG_001; Photobacterium; Desulforio; Butyricicoccus*
Down-regulated in both BPS and FMT-BPS (5)	*Desulfovibrio; Faecalibaculum; Odoribacter; Rikenella; Tritrichomonas*
“Rescued” by AOS (1)	*Parabacteroides*
Down-regulated in AOS+BPS vs BPS (56)	*Lachnospiraceae_NK4A136_group; Romboutsia; Blautia; Sphingomonas; Vicinamibacteraceae; Clostridium_sensu_stricto_1; RB41; Enterorhabdus; Turicibacter; Rubrobacter; TRA3_20; Legionella; Bacteriap25; Pontibacter; MND1; Steroidobacter; Subgroup_7; Microvirga; Acidibacter; Pleomorphomonas; Asticcacaulis; Subgroup_10; Terrimonas; Paenibacillus; Devosia; Skermanella; Nitrospira; Rhizobium; [Eubacterium] coprostanoligenes_group; Azospirillum; KD4_96; Massilia; SC_I_84; Bryobacter; Caulobacter; Delftia; Pedosphaeraceae; Nitrosospira; Rokubacteriales; Dongia; Nordella; S085; Blastococcus; PLTA13; Altererythrobacter; Flavisolibacter; Mesorhizobium; 67_14; Chujaibacter; AKAU4049; Dyadobacter; Flavitalea; Variovorax; Ramlibacter; Anaerocolumna; Kaistia*
Up-regulated in both BPS and FMT-BPS (5)	*Family_XIII_AD3011_group; NK4A214_group; Rikenellaceae_RC9_gut_group; Tyzzerella; UCG-009*
Suppressed by AOS (7)	*[Eubacterium] nodatum_group; [Eubacterium] siraeum_group; [Eubacterium] ventriosum_group; Clostridia_UCG_014; Ellin6055; Mucispirillum; RF39*

Table S4. GO-BP analysis of genes with altered expression in BPS and FMT-BPS treated oocytes.

Alteration	Pathway	Genes
**Up-regulated**	• striated muscle tissue development	Popdc3; Akirin1; Sap30; Myocd; Nox4; Bmp7; Gpc1; Wnt2; Actn2; Gata4
	• muscle tissue development	Popdc3; Akirin1; Sap30; Myocd; Nox4; Bmp7; Gpc1; Wnt2; Actn2; Gata4
	• regulation of protein serine; threonine kinase activity	Dusp1; Map2k2; Ccnl1; Fgd2; Myocd; Gab1; Tead1; Nox4; Bmp7
	• negative regulation of protein phosphorylation	Socs1; Dusp1; Chrna9; Klhl31; Ppm1e; Myocd; Bmp7; Dmtn
	• negative regulation of phosphorylation	Socs1; Dusp1; Chrna9; Klhl31; Ppm1e; Myocd; Bmp7; Dmtn
	• reproductive structure development	Arid5b; Etnk2; Vcam1; Myocd; Gab1; Bmp7; Wnt2; Gata4
	• reproductive system development	Arid5b; Etnk2; Vcam1; Myocd; Gab1; Bmp7; Wnt2; Gata4
	• ERK1 and ERK2 cascade	Styx; Dusp1; Map2k2; Chrna9; Nox4; Epha7; Gata4
	• regulation of ERK1 and ERK2 cascade	Styx; Dusp1; Chrna9; Nox4; Epha7; Gata4
	• regulation of stress-activated MAPK cascade	Dusp1; Map2k2; Klhl31; Fgd2; Gab1
	• regulation of stress-activated protein kinase signaling cascade	Dusp1; Map2k2; Klhl31; Fgd2; Gab1
	• stress-activated MAPK cascade	Dusp1; Map2k2; Klhl31; Fgd2; Gab1
	• calcium-mediated signaling	Ccr9; Nmur1; Vcam1; Dmtn
	• regulation of leukocyte migration	C1qbp; Dusp1; Akirin1; Capn1
	• regulation of cardiac muscle cell apoptotic process	Myocd; Capn1; Gata4
	• regulation of striated muscle cell apoptotic process	Myocd; Capn1; Gata4
	• cardiac muscle cell apoptotic process	Myocd; Capn1; Gata4
	• striated muscle cell apoptotic process	Myocd; Capn1; Gata4
	• autophagy of nucleus	Atg9b; Atg3
	• activation of phospholipase C activity	Nmur1; Arhgap6
**Down-regulated**	• organic anion transport	Stard10; Slc4a3; Pla2g1b; Slc16a4; Cftr; Slc16a5; Lrp2; Abcb11; Emb; Slc6a12; Slc39a8
	• organic hydroxy compound metabolic process	Dio3; Epas1; Fgl1; Cftr; Akr1c21; Disp3; Abcb11; Npr1; Pnmt; Fmo5
	• carboxylic acid transport	Stard10; Pla2g1b; Slc16a4; Slc16a5; Lrp2; Abcb11; Emb; Slc6a12
	• axonogenesis	Chl1; Slit3; Tnr; Sema3a; Dvl1; Lrp2; Emb; Gap43
	• monocarboxylic acid transport	Stard10; Pla2g1b; Slc16a4; Slc16a5; Abcb11; Emb; Slc6a12
	• axon guidance	Chl1; Slit3; Sema3a; Dvl1; Lrp2; Emb; Gap43
	• leukocyte cell-cell adhesion	Ccl2; Fgl1; Tnfrsf21; Gm5150; Il12rb1; Btnl2; Slc39a8
	• fatty acid metabolic process	Pla2g1b; Cyp2a4; Akr1c21; Abcb11; Eif2ak3; Fasn; Cyp2d26
	• regulation of cell-cell adhesion	Ccl2; Fgl1; Tnfrsf21; Tnr; Gm5150; Il12rb1; Btnl2
	• positive regulation of cytokine production	Polr3g; Ccl2; Il12rb1; Il17b; Nfam1; Btnl2; Eif2ak3
	• positive regulation of cell activation	Ccl2; Igll1; Cftr; Gm5150; Il12rb1; Ttbk1; Btnl2
	• antimicrobial humoral response	Ccl2; Pla2g1b; Reg3g; Wfdc21; Cst9; Rnase6
	• heart contraction	Epas1; Slc4a3; Sema3a; Bves; Trdn; Glp1r
	• heart process	Epas1; Slc4a3; Sema3a; Bves; Trdn; Glp1r
	• steroid metabolic process	Fgl1; Cftr; Akr1c21; Disp3; Abcb11; Fmo5
	• neuron death	Chl1; Pm20d1; Kcnb1; Tnfrsf21; Rilpl1; Glp1r
	• drug metabolic process	Cyp2a4; Abcb11; Fmo5; Cyp2d26
	• axon extension	Slit3; Tnr; Sema3a; Dvl1
	• cholesterol metabolic process	Fgl1; Cftr; Disp3; Fmo5
	• phenol-containing compound metabolic process	Dio3; Epas1; Npr1; Pnmt

Table S5. Primers used for RT-qPCR.

Genes	Gene Bank accession No.	Primer sequence(5'-3')	Tm (ºC)	Product size (bp)
Tnr	NM_022312.3	F: GGCTGGAGGTGACTACAGAAA<br>R: GAAGACCATAGGCTGTTCCTTG	60	101
Sema3a	NM_001243072.1	F: GGCTGGTTCACTGGGATTG<br>R: CCGTTTGCATAGTTTGCTCTGG	60	71
Dvl1	NM_001356381.1	F: CCTCCTTCCAACCAAATGTTGC<br>R: CTGCTCAGTACAGTAGATGCAC	60	199
Slc39a8	NM_001135149.1	F: GCCAAGCTCATGTACCTGTCT<br>R: AAGATGCCCCAATCGCCAA	60	122
Cftr	NM_021050.2	F: CTGGACCACACCAATTTTGAGG<br>R: GCGTGGATAAGCTGGGGAT	60	162
Dusp1	NM_013642.3	F: GTTGTTGGATTGTCGCTCCTT<br>R: TTGGGCACGATATGCTCCAG	60	129
Gapdh	NM_001289726.1	F: CCTTCCGTGTTCCTACCC<br>R: CAACCTGGTCCTCAGTGTAG	60	150
Arhgap6	NM_001287530.1	F: CTCGGCCAAGGGATTCTCC<br>R: AATGAAGCGGCTTACTGTGAG	60	128
Atg3	NM_026402.3	F: ACACGGTGAAGGGAAAGGC<br>R: TGGTGGACTAAGTGATCTCCAG	60	130
Atg9b	NM_001002897.3	F: CCATCCCACAATGATACACACC<br>R: CCTCTAGCCGTTCATAGTCCT	60	164
Nox4	NM_015760.5	F: GAAGGGGTTAAACACCTCTGC<br>R: ATGCTCTGCTTAAACACAATCCT	60	145
